# Construction of a Mitochondria‐Related Gene Diagnostic Model Based on Integrated Multiomics Data and Functional Validation of ANK2 as a Key Regulator in Colorectal Cancer

**DOI:** 10.1155/ijog/9306920

**Published:** 2026-01-31

**Authors:** Xiangyu Ding, Huanhuan Wu, Jiyuan Yang, Han Song, Jianhui Guo, Xudong Wang, Xiaopeng Zhang

**Affiliations:** ^1^ Department of Laboratory Medicine, Affiliated Hospital of Nantong University, Medical School of Nantong University, Nantong, Jiangsu, China, ntu.edu.cn; ^2^ Department of Laboratory Medicine, People′s Hospital of Haimen District, Nantong, Jiangsu, China

**Keywords:** ANK2, colorectal cancer, early diagnostic model, machine learning, mitochondria-related genes, multiomics data

## Abstract

Colorectal cancer (CRC) is one of the most common malignancies of the digestive tract globally, characterized by high incidence, difficulty in early diagnosis, and poor prognosis. Traditional screening methods have limitations in sensitivity and specificity, thus necessitating the development of novel, efficient molecular diagnostic approaches. Recent studies have highlighted the crucial role of mitochondrial dysfunction in the initiation and progression of various cancers, suggesting that mitochondria‐related genes (MRGs) could serve as promising diagnostic targets for CRC. In this study, we integrated transcriptomic data from 1174 samples across The Cancer Genome Atlas (TCGA) and multiple Gene Expression Omnibus (GEO) public datasets (GSE21510, GSE44076, and GSE9348) and combined it with MRG data from the MitoCarta3.0 database for a systematic analysis of differentially expressed genes (DEGs). Using LASSO regression and SVM‐RFE, two machine learning algorithms, we identified eight key MRGs (ABCG2, ANK2, MACC1, PMAIP1, SLC22A5, SLC25A34, ACAT1, and PDK4) and constructed an early diagnostic model for CRC. Receiver operating characteristic (ROC) curve analysis confirmed the diagnostic efficacy of the model. Gene interaction networks were constructed using GeneMANIA, demonstrating the potential synergistic roles of these genes in regulating cellular metabolism, drug efflux, and immune modulation. CIBERSORT immune cell infiltration analysis revealed significant correlations between these genes and various immune cell subtypes, including T cells, macrophages, and dendritic cells. Further integration of single‐cell RNA sequencing data (GSE245552) identified the specific expression patterns of the diagnostic model genes across different cell types. Additionally, we conducted an in‐depth investigation of the ANK2 gene. Immunohistochemistry (HPA database), qRT‐PCR, and western blotting confirmed the significantly low expression of ANK2 in CRC tissues and cell lines. Moreover, TUNEL and angiogenesis assays showed that overexpression of ANK2 significantly promoted cell apoptosis and inhibited angiogenesis, suggesting that ANK2 may function as a key tumor suppressor in CRC. In conclusion, this study proposes and validates a CRC diagnostic model based on differentially expressed mitochondrial genes. We systematically explored the molecular mechanisms and immune microenvironment correlations of the model and confirmed the biological effects through single‐cell and molecular biology experiments. Notably, we highlight the potential regulatory role of ANK2 in the progression of CRC. This research provides theoretical support and new directions for early screening, diagnostic biomarker identification, and targeted therapy strategies for CRC.

## 1. Introduction

Colorectal cancer (CRC) is one of the most prevalent malignant tumors worldwide and poses a significant threat to public health [1]. According to the Global Cancer Statistics 2020, approximately 1.9 million new CRC cases and 940,000 related deaths were reported globally, making CRC the second leading cause of cancer‐related mortality [2]. Common screening methods for CRC include fecal occult blood tests, while colonoscopy is considered the gold standard. Other diagnostic approaches include imaging techniques such as transrectal ultrasound (TRUS), abdominal ultrasound, computed tomography (CT), and magnetic resonance imaging (MRI) [3]. However, these methods are often effective only for detecting advanced, localized lesions.

In recent years, tumor biomarkers have gained increasing attention in cancer diagnostics and treatment. An ideal biomarker should exhibit high specificity and sensitivity, particularly for early stage detection [4]. Although various therapeutic strategies including surgical resection, radiotherapy, and immunotherapy are available for CRC, early diagnosis significantly improves patient prognosis and survival rates [5]. Therefore, developing diagnostic models with high sensitivity and specificity is crucial to enhancing clinical outcomes and quality of life for CRC patients.

Mitochondria are vital organelles in eukaryotic cells and the only cytoplasmic organelles that contain their own genome [6]. Often referred to as the “powerhouse” of the cell, mitochondria play essential roles in cellular energy metabolism, reactive oxygen species (ROS) generation, and regulation of apoptosis [7]. Given their central role in cell physiology, mitochondrial dysfunction has been increasingly implicated in tumorigenesis and cancer progression [8]. Despite growing interest in mitochondria‐related genes (MRGs) in various cancers, research specifically focusing on their roles in CRC remains limited.

In this study, we integrated transcriptomic data from CRC samples in TCGA and multiple GEO datasets and combined these with a curated list of MRGs. Utilizing two machine learning algorithms, least absolute shrinkage and selection operator (LASSO) regression and support vector machine–recursive feature elimination (SVM‐RFE), we constructed a novel diagnostic model for CRC composed of eight MRGs: ATP‐binding Cassette Subfamily G Member 2 (ABCG2), ANK2, MACC1, PMAIP1, SLC22A5, SLC25A34, acetyl‐CoA Acetyltransferase 1 (ACAT1), and pyruvate dehydrogenase Kinase 4 (PDK4). To validate the model, we applied a comprehensive array of methods including CIBERSORT for immune cell infiltration analysis, ROC curve analysis, GeneMANIA for gene interaction networks, and single‐cell annotation. Additionally, given the limited research on ANK2 in CRC, we conducted further molecular and cellular functional assays to explore its role in CRC initiation and progression.

## 2. Methods

### 2.1. Dataset Collection and Processing

The RNA sequencing data of CRC samples were sourced from the TCGA database and the GEO database (GSE21510, GSE44076, and GSE9348). The data were normalized and integrated using the R packages “limma” and “sva,” resulting in a total of 938 tumor samples and 236 normal samples. The mitochondrial‐associated gene dataset was obtained from the MitoCarta3.0 database (https://www.broadinstitute.org/mitocarta/mitocarta30-inventory-mammalian-mitochondrial-proteins-and-pathways).

### 2.2. Differential Gene Expression Analysis

Differential gene expression analysis was performed to assess the gene expression differences between CRC and normal tissues. The threshold for identifying differentially expressed genes (DEGs) was set to |logFC| ≥ 2 and adjusted *p* value < 0.05. Volcano plots and heatmaps were generated using the R packages “ggplot2” and “pheatmap.”

### 2.3. Enrichment Analysis

GO (Gene Ontology) and KEGG pathway enrichment analyses were conducted using the R packages “org.Hs.eg.db” and “EnrichPlot.” Each GO annotation consists of a gene and its corresponding GO term, focusing on three main categories: molecular function (MF), biological process (BP), and cellular component (CC). The KEGG database, established by the Kanehisa Laboratory at Kyoto University in 1995, is a vital bioinformatics resource used to integrate and interpret large‐scale molecular datasets generated by genomic sequencing and other high‐throughput technologies. A significance threshold of adjusted *p* < 0.05 was applied to identify statistically significant candidate functions. Additionally, GSEA (Gene Set Enrichment Analysis) was used to further explore potential biological characteristics. GSEA is a gene set–based analysis method that evaluates the enrichment of predefined gene sets in samples, helping to identify genomic patterns associated with specific BPs or pathways. The strength of GSEA lies in its ability to uncover new BPs and identify key pathways associated with disease, providing valuable insights for future experimental research and clinical applications. These analyses offer essential theoretical support for optimizing CRC clinical diagnostics and treatment strategies.

### 2.4. Mitochondrial Gene Selection for CRC Diagnosis Using LASSO and SVM Regression

LASSO and SVM‐RFE regression are widely used algorithms for variable selection, particularly in the construction of tumor diagnosis and prognosis models. In this study, both algorithms were employed to select key mitochondrial genes for CRC diagnosis, and the intersection of the results was considered. The diagnostic capability of the key CRC genes was assessed using the area under the receiver operating characteristic curve (AUC).

### 2.5. Gene Interaction Analysis of CRC Mitochondrial Gene Diagnostic Model

To elucidate the potential BPs and pathways associated with the key genes, a coexpression network of the key genes and their similar genes was constructed using GeneMANIA (http://www.genemania.org/). This network analysis allowed for a deeper understanding of the intrinsic relationships between genes. Additionally, the PerformanceAnalytics R package was used to further analyze the synergistic effects of the key genes in CRC.

### 2.6. Immune Cell Infiltration Analysis in CRC Patients

CIBERSORT is a widely used tool based on linear support vector regression to deconvolute immune cell subtype expression matrices. It is currently one of the most commonly employed methods for immune cell infiltration estimation. xCell, another tool for immune cell infiltration estimation, constructs a unique gene expression profile to provide a more detailed classification of immune cells in the tumor microenvironment (TME), especially for rare or incompletely characterized immune cell types, demonstrating higher sensitivity and accuracy. By combining the results from xCell and CIBERSORT, a comprehensive evaluation of the immune cell composition and functional status in the CRC immune microenvironment was conducted.

### 2.7. Single‐Cell RNA Sequencing (ScRNA‐Seq) Data Collection and Processing

ScRNA‐seq allows for a comprehensive understanding of cellular heterogeneity within the TME at the single‐cell level, further revealing the mechanisms underlying tumor progression. In this study, we downloaded the public scRNA‐seq dataset GSE245552, which includes 130 samples from 18 CRC patients, totaling over 160,000 single‐cell data points. To further investigate the immune microenvironment and tumor cell characteristics in CRC, data from 15 CRC tumor tissue samples and 4 adjacent normal tissue samples were selected for subsequent analysis. Data processing was performed using the Seurat R package, a widely used tool for scRNA‐seq data analysis. Quality control was conducted to filter cells meeting the following criteria: nFeature_RNA > 50 and percent.mt < 5. Here, nFeature_RNA represents the number of genes expressed in each cell, and percent.mt indicates the proportion of mitochondrial genes, with a higher proportion typically reflecting poor cell quality or cell death. By applying these filtering conditions, low‐quality or abnormal cells were removed, ensuring data accuracy. Next, the data were normalized using the Seurat package, and the 1500 most variable genes were selected for principal component analysis (PCA), which effectively reduced the dimensionality of the data, preserving the most representative features and minimizing noise. PCA is a common dimensionality reduction technique that helps extract key information from high‐dimensional gene expression data, preparing the data for subsequent clustering analysis. To further explore cellular diversity, t‐distributed stochastic neighbor embedding (t‐SNE) was used for dimensionality reduction, and cells were classified into different clusters based on their gene expression patterns. t‐SNE is a nonlinear dimensionality reduction method that maps high‐dimensional data to a two‐dimensional space, facilitating visualization and clustering. Each cell cluster represents a group of cells with similar gene expression patterns. Using the singleR R package, cell types within each cluster were annotated by comparing the data with predefined reference datasets. This approach automatically assigns appropriate cell type labels to each cluster based on similarity to the reference data, providing valuable insights into the functional interpretation of the cell populations.

### 2.8. Apoptosis Scoring

Apoptosis, a programmed cell death mechanism, plays a critical role in tumor initiation, progression, and therapeutic responses. To assess apoptosis activity in CRC cells, the AUCell package was used to score apoptosis gene sets in single‐cell data. AUCell is a method based on GSEA that evaluates the activity level of specific gene sets in each cell. Changes in apoptosis scores can reveal mechanisms of apoptosis evasion in cancer cells, providing potential therapeutic targets for CRC.

### 2.9. Cell Lines and Cell Culture

Human CRC cell lines (NCM460, SW620, RKO, HCT1116, SW1116, and LoVo) were obtained from the Chinese Academy of Sciences (Shanghai, China). Each cell line was authenticated by short tandem repeat (STR) analysis, and cells were cultured for less than 6 months after authentication. All cell lines were maintained in RPMI 1640 medium (Thermo, New York, United States), supplemented with 10% fetal bovine serum (FBS) (Gibco, California, United States), and incubated at 37°C in a 5% CO_2_ environment. Human umbilical vein endothelial cells (HUVECs) were obtained from the Cell Bank of the Chinese Academy of Sciences (Beijing, China). The cells were confirmed as endothelial cells by immunocytochemical staining using anti‐Factor VIII antibody (BS‐0434R, Bioss, China). HUVECs were cultured in high‐glucose DMEM supplemented with 10% (v/v) FBS and maintained at 37°C in a humidified incubator with 5% CO_2_ and 95% air.

### 2.10. RNA Extraction and qRT‐PCR Detection

Total RNA was extracted using the RNA isolation reagent (Vazyme, Jiangsu, China) according to the manufacturer′s instructions. The extracted RNA was reverse‐transcribed into cDNA, and quantitative PCR (qPCR) was performed for quantification. Data collection was carried out using LightCycler 480 SYBR Green I Master (Vazyme, Jiangsu, China). GAPDH was used as the reference gene to ensure consistent sample loading. PCR primers were designed by China Bioengineering.

### 2.11. Western Blotting

Proteins were separated by 12% SDS‐PAGE and transferred to a PVDF membrane. After blocking with 3% skim milk for 2 h at room temperature, the membrane was washed three times with TBST. The primary antibody (1:5000) was incubated overnight at 4°C, followed by three washes with TBST. The secondary antibody (1:10,000) was incubated at room temperature for 2 h, and the membrane was washed three times with TBST. Protein bands were visualized and photographed.

### 2.12. Construction of ANK2 Overexpression and Knockdown Models

LoVo cells (human CRC cells) were cultured in antibiotic‐free complete medium to 50%–70% confluence. Overexpression plasmids of ANK2 were mixed with Opti‐MEM and incubated with transfection reagents to form complexes, which were then added to the cells and incubated at 37°C for 6–8 h before replacing with fresh medium. After 24 h of transfection, qPCR was performed to verify the overexpression efficiency.

For knockdown, siRNA and transfection reagents were diluted in serum‐free medium. LoVo cells were transfected with siRNA using Lipofectamine 3000, and after 24 h, RNA was extracted for qPCR analysis to verify knockdown efficiency.

### 2.13. TUNEL Assay

LoVo cells were seeded in 6‐well plates and cultured until the cell density reached approximately 50%–70%. The ANK2 overexpression plasmid (ANK2‐OE), ANK2‐specific siRNA (ANK2‐KD), and nontargeting siRNA (ANK2‐NK) were transfected into LoVo cells using a transfection reagent, followed by continued culture for 24 h. The transfected LoVo cells were allowed to adhere and grow in the 6‐well plates until they reached an appropriate state. After stable growth, the culture medium was aspirated, and the cells were washed with PBS for 3 min, repeated three times. The cells were then fixed with 4% paraformaldehyde for 30 min. Subsequently, the cells were washed again with PBS for 3 min, repeated three times. Next, the cells were incubated with PBS containing 0.3% Triton X‐100 at room temperature for 10 min, followed by another PBS wash for 3 min, repeated three times. Under dark conditions, the TUNEL apoptosis reagent working solution was added, and the plate was wrapped in aluminum foil and incubated at 37°C for 1 h. The working solution was then washed off with PBS. Finally, 5 *μ*L of antifade mounting medium was applied to a glass slide, and the cell‐covered slide was positioned properly. Fluorescence microscopy was performed using an excitation wavelength range of 450–500 nm, and the green fluorescent signals were recorded.

### 2.14. Angiogenesis Assay

LoVo cells were cultured to an appropriate density. The ANK2 overexpression plasmid (ANK2‐OE), ANK2‐specific siRNA (ANK2‐KD), and nontargeting siRNA (ANK2‐NK) were transfected into LoVo cells using a transfection reagent, followed by continued incubation for 24 h. The supernatant medium from different groups was collected, centrifuged at 1000 rpm for 10 min to remove cell debris, filtered through a 0.22 *μ*m filter, and stored at 4°C as conditioned medium. Autoclaved 96‐well plates and pipette tips were placed at −20°C overnight for freezing. Matrigel was thawed at 4°C, diluted at a 1:1 ratio, and evenly coated onto the 96‐well plates. The plates were then incubated at 4°C for 2 h to ensure uniform distribution of the Matrigel. Subsequently, the plates were transferred to a 37°C incubator for 1 h to allow complete solidification of the Matrigel. HUVEC cells were seeded into the 96‐well plates and treated with conditioned medium from different groups. The cells were then further cultured in the incubator. After 24 h of incubation, the formation of vascular‐like structures by HUVEC cells in the Matrigel was observed. Tubular structures formed by HUVEC cells were examined under a microscope and photographed. The differences in angiogenesis were compared among the different groups (control, ANK2‐OE, ANK2‐KD, and ANK2‐NK).

### 2.15. Statistical Analysis

Statistical analysis was performed using R (4.4.1), ImageJ, and GraphPad Prism 9.5.0. Intergroup comparisons were conducted using independent *t*‐tests. A significance level of *p* < 0.05 was considered statistically significant.

## 3. Results

### 3.1. Technical Roadmap (Figure 1)

**Figure 1:**
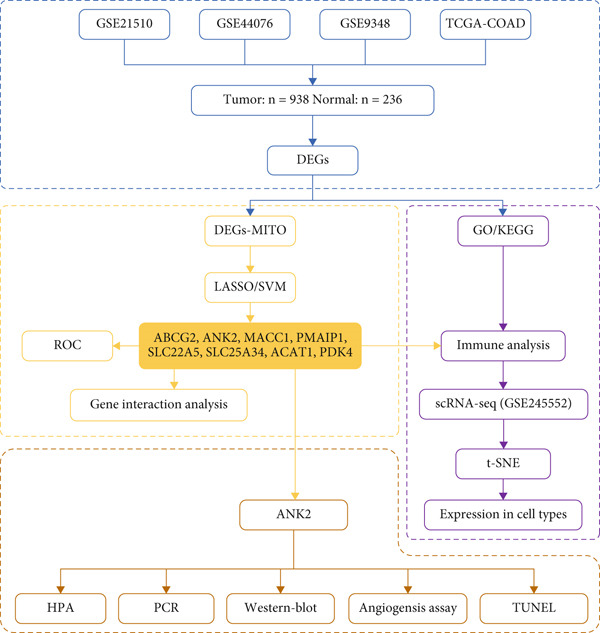
Technology roadmap.

### 3.2. Identification and Enrichment Analysis of DEGs

Datasets GSE21510, GSE44076, GSE9348, and TCGA‐COAD were downloaded and merged using the limma and sva packages for normalization. We plotted PCA diagrams before and after batch correction to observe the effect of batch removal (File S7). A total of 236 normal tissue samples and 938 CRC tissue samples were obtained. Differential expression analysis was then performed on the integrated dataset, identifying 7422 DEGs, which were visualized using heatmaps and volcano plots (Figures 2a,b).

Figure 2Difference analysis and enrichment analysis. (a) Heatmap of differential genes. (b) Volcano map of differential genes. (c) Enrichment of the Top 30 GOs (ranked by *p* value). (d) Enrichment of the Top 30 KEGGs.

**Figure:**
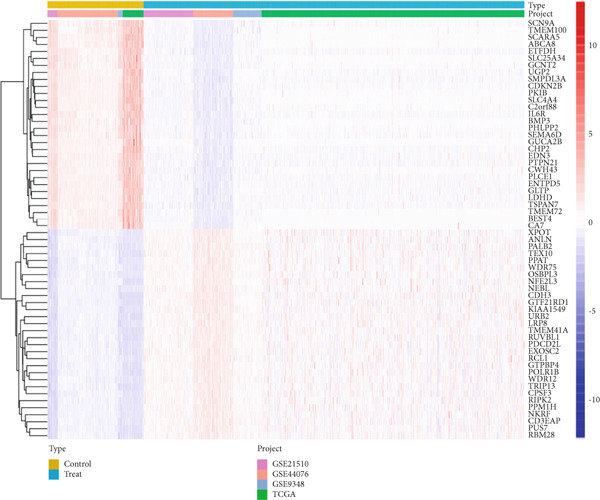
(a)

**Figure:**
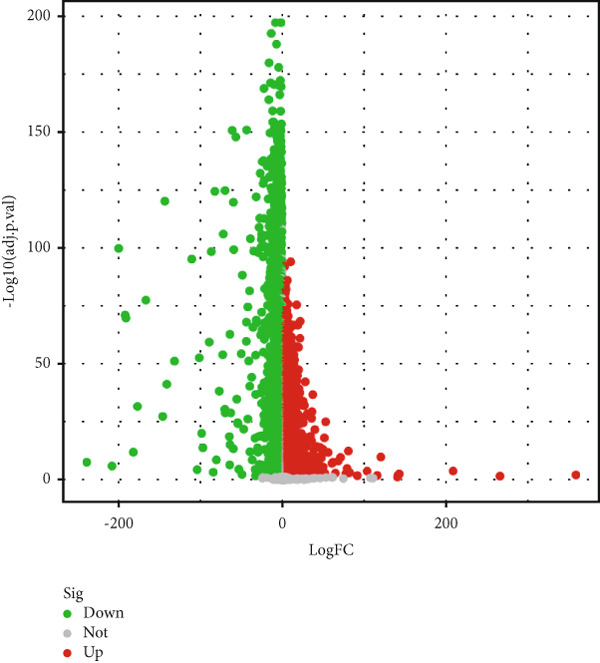
(b)

**Figure:**
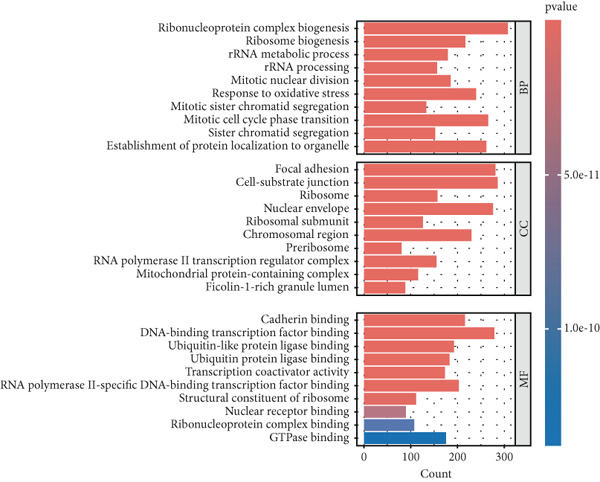
(c)

**Figure:**
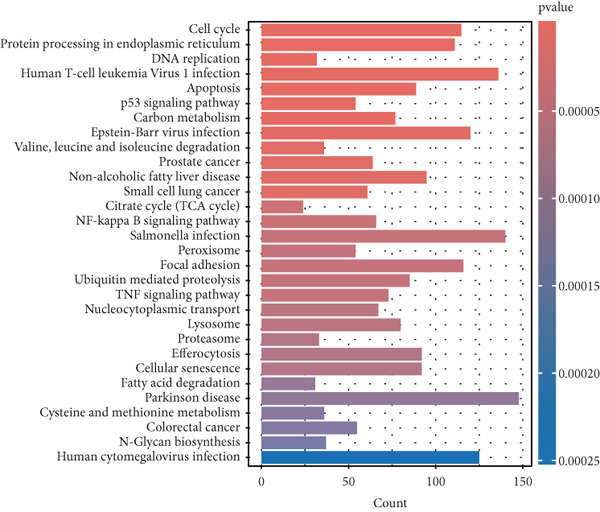
(d)

Subsequently, a GO enrichment analysis was performed on the DEGs, which revealed their involvement in BP such as protein localization to organelles, sister chromatid separation, mitotic cell cycle phase transition, mitotic sister chromatid separation, and response to oxidative stress. CC was mainly associated with ficolin‐1‐rich granule lumen, complexes containing mitochondrial proteins, RNA Polymerase II transcription regulation complexes, preribosomes, and chromosomal regions. MF included GTPase binding, ribonucleoprotein complex binding, nuclear receptor binding, ribosome structural component, and RNA Polymerase II–specific DNA‐binding transcription factor binding (Figure 2c).

These results highlight the relationship between the DEGs and key processes such as the cell cycle, metabolism, immune microenvironment, and tumor metastasis. KEGG pathway analysis further identified enriched pathways related to the cell cycle, protein processing in the endoplasmic reticulum, DNA replication, HTLV‐1 infection, apoptosis, p53 signaling pathway, and carbon metabolism (Figure 2d).

GSEA enrichment analysis of the DEGs revealed significant enrichment of pathways like necroptosis, cell cycle, DNA replication, and Wnt signaling pathway in CRC tissues, with statistical significance (File S1). These analyses collectively provide a “molecular activity map” of CRC cells, outlining the progression from DNA replication initiation to cell cycle dysregulation, programmed necrosis regulation, and sustained Wnt signaling activation. This suggests that abnormalities in these pathways play a core role in tumorigenesis, progression, and immune microenvironment formation.

### 3.3. Construction of a Mitochondria‐Related Differential Gene Diagnostic Model for CRC

Mitochondrial gene expression abnormalities are closely linked to the metabolic reprogramming and immune microenvironment regulation of tumors [9]. These abnormalities are also associated with tumor prognosis, therapeutic response, and molecular subtyping, making them potential molecular biomarkers for precise cancer diagnosis. Therefore, we used a mitochondrial gene set from the MitoCarta3.0 database (File S2) to further filter the DEGs, ultimately identifying 1020 mitochondria‐related differential genes.

We then employed LASSO regression and SVM‐RFE methods for further analysis. Using LASSO regression, we identified 75 genes (Figure 3a), while the SVM‐RFE algorithm selected 31 feature genes (Figure 3b). Notably, eight genes were found to overlap between the two methods (Figure 3c), namely, ABCG2, ANK2, MACC1, PMAIP1, SLC22A5, SLC25A34, ACAT1, and PDK4.

Figure 3Machine learning intersects. (a) Seventy‐five characteristic genes were obtained by LASSO regression analysis. (b) Thirty‐one characteristic genes were obtained by SVM analysis. (c) Overlapping genes of two machine learning algorithms were obtained. (d) ROC analysis of diagnostic model genes. (e) Boxplot of differential expression of each gene in cancerous tissue versus adjacent tissue across all samples ( ^∗^ *p* < 0.05;  ^∗∗^ *p* < 0.01;  ^∗∗∗^ *p* < 0.001).

**Figure:**
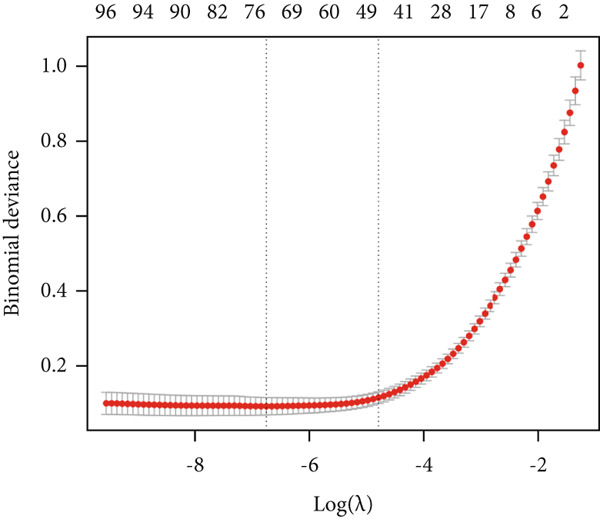
(a)

**Figure:**
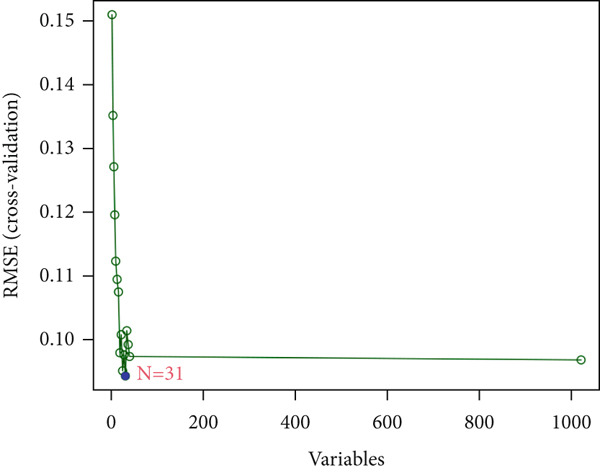
(b)

**Figure:**
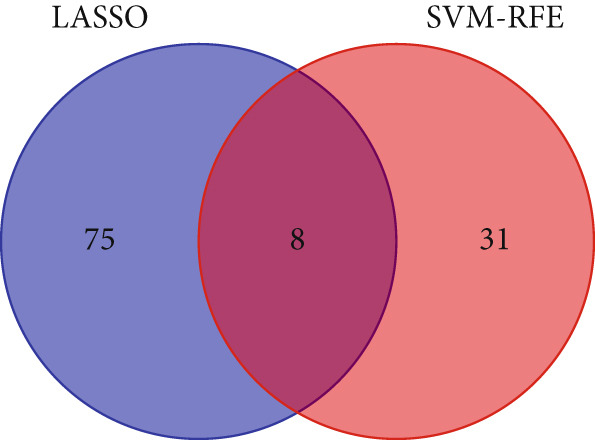
(c)

**Figure:**
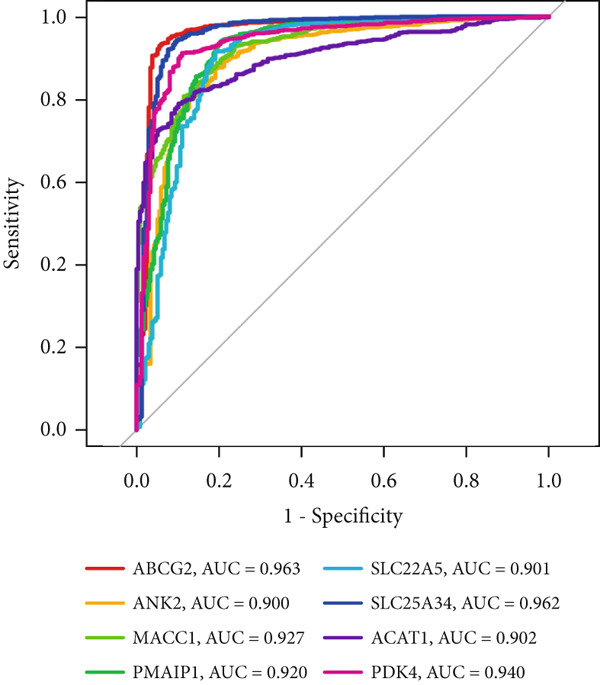
(d)

**Figure:**
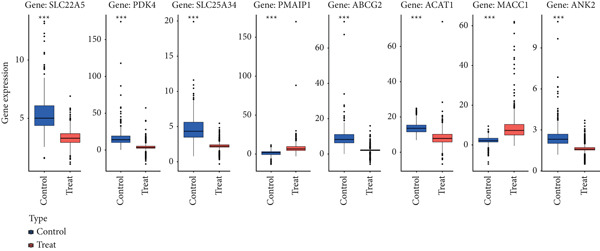
(e)

To evaluate the diagnostic accuracy of these eight genes as biomarkers, we plotted ROC curves, revealing that the AUC values for all genes exceeded 0.9 (Figure 3d), indicating a highly effective diagnostic model. Subsequently, we analyzed the differential expression of these eight genes across all samples and found that ABCG2, ANK2, SLC22A5, SLC25A34, ACAT1, and PDK4 were downregulated in CRC, whereas MACC1 and PMAIP1 were upregulated in CRC (Figure 3e).

### 3.4. Gene Interaction in the Diagnostic Model and Correlation With Immune Cell Infiltration

To investigate the interactions among key genes, we constructed a gene interaction network using GeneMANIA. This network revealed that genes may interact through coexpression or shared protein domains, playing crucial roles in various BPs such as tumor drug resistance, metabolic reprogramming, apoptosis regulation, and signal pathway modulation (Figure 4a). Notably, genes associated with PDK4 suggest its potential involvement in regulating glycolysis and affecting mitochondrial function. ACAT1 is linked to fatty acid metabolism, while ABCG2 influences drug efflux, potentially contributing to tumor resistance mechanisms. PMAIP1 regulates apoptosis and is associated with cancer, ANK2 is related to cardiac function and cytoskeletal stability, and SLC22A5 and SLC25A34 are involved in solute transport and energy metabolism (File S1). These results highlight the synergistic roles of these genes in tumor progression and provide new molecular targets for early cancer diagnosis.

Figure 4There are synergistic effects between diagnostic model genes that are associated with a variety of biological processes and immune cell infiltration. (a) GeneMANIA gene interaction diagram: The inner circle is the eight key genes for diagnosis, and the outer circle is the Top 20 genes with the strongest correlation. (b) Correlation of gene expression in CRC. (c) The CIBERSORT algorithm calculates the immune infiltration of the diagnostic model genes in CRC, with the correlation between immune cells in the lower left corner and the correlation between each type of immune cell in the upper right corner ( ^∗^ *p* < 0.05;  ^∗∗^ *p* < 0.01;  ^∗∗∗^ *p* < 0.001).

**Figure:**
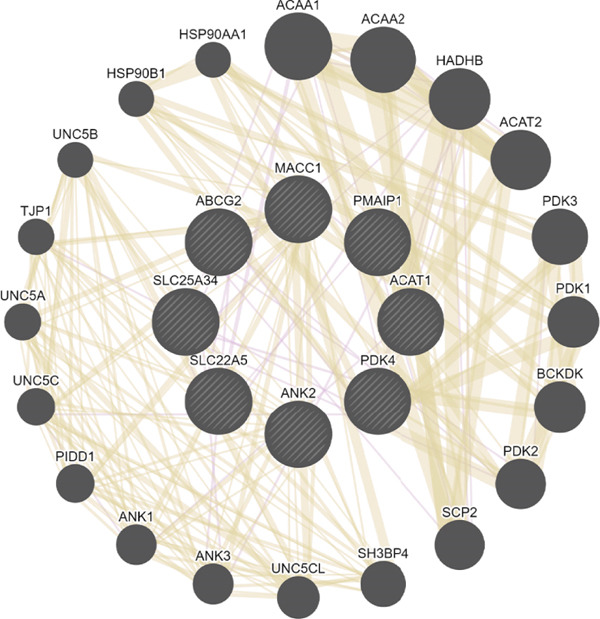
(a)

**Figure:**
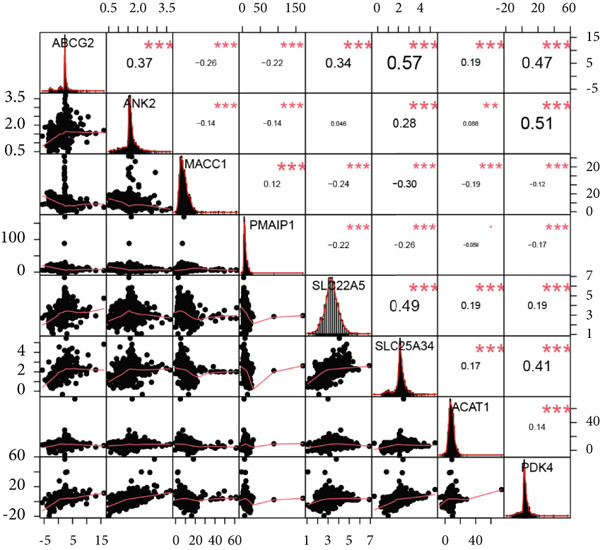
(b)

**Figure:**
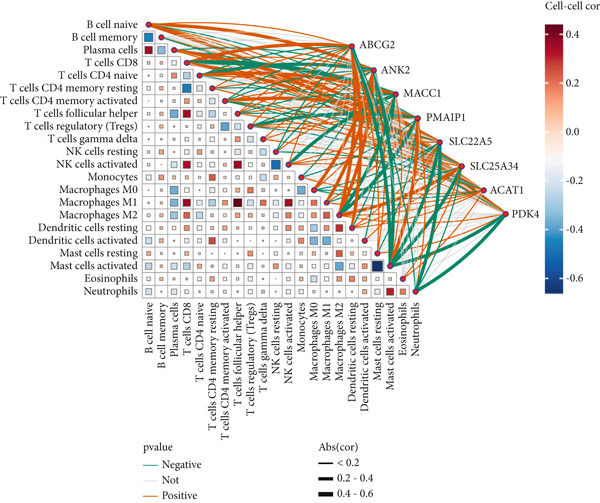
(c)

Next, we used the PerformanceAnalytics package to analyze the correlations between gene expression levels in the diagnostic model within CRC. The results showed significant correlations between nearly all pairs of these eight key genes, except for ANK2 and SLC22A5 and PMAIP1 and ACAT1 (Figure 4b), further supporting their cooperative roles in CRC. We conducted GO analysis and KEGG analysis on these eight core genes (File S8A,B). Core genes are significantly enriched in BPs related to metabolism, transport, and stress response. Metabolism‐related processes include acetyl‐CoA biosynthesis and metabolism, acyl‐CoA biosynthesis and metabolism, thioester biosynthesis and metabolism, fatty acid oxidation, lipid oxidation, glucose metabolism, and regulation of carbohydrate metabolism. Transport‐related processes involve xenobiotic transport, vascular transport, blood–brain barrier transmembrane transport, and plasma membrane efflux. Stress and regulatory processes encompass hunger response, cellular starvation response, detoxification, sulfur compound biosynthesis, and amide biosynthesis. Notably, the enrichment results for acyl‐CoA metabolic processes (*p* = 0.001) and the regulation of glucose metabolism (*p* = 0.002) are statistically significant (*p* < 0.05). KEGG pathway analysis indicates that differential molecules are mainly associated with metabolic pathways, resistance pathways, and other key pathways. Metabolic pathways include terpenoid backbone biosynthesis, butanoate metabolism, glyoxylate and dicarboxylate metabolism, folate transport and metabolism, tryptophan metabolism (*p* = 0.02), fatty acid degradation (*p* = 0.03), fat digestion and absorption (*p* = 0.04), pyruvate metabolism, valine–leucine–isoleucine degradation, lysine degradation, and fatty acid metabolism. Resistance pathways involve antifolate resistance and platinum drug resistance, while other pathways include ABC transporters and apoptosis across multiple species. The enrichment results for tryptophan metabolism, fatty acid degradation, and fat digestion and absorption are all statistically significant (*p* < 0.05). These results suggest that core differential molecules may regulate physiological or pathological processes in the study system through modulating energy metabolism, such as fatty acid and glucose metabolism, transmembrane transport of substances, and drug resistance–related pathways, providing key functional directions for subsequent mechanistic validation.

Building upon the earlier enrichment analysis, which indicated the involvement of DEGs in immune‐related pathways, we used the CIBERSORT algorithm to calculate immune cell infiltration based on the diagnostic model genes in CRC. The results revealed significant correlations between these eight genes and multiple immune cell types. Among the most notable associations, ABCG2 was positively correlated with CD8+ T cells, M2 macrophages, and resting dendritic cells and negatively correlated with activated mast cells; ANK2 was positively correlated with naive B cells and macrophages and negatively correlated with naive CD4+ T cells and activated mast cells; MACC1 was positively correlated with naive CD4+ T cells and negatively correlated with CD8+ T cells; PMAIP1 showed a positive correlation with activated mast cells and memory‐activated CD4+ T cells and a negative correlation with Tregs and M2 macrophages; SLC22A5 was positively correlated with plasma cells and naive B cells and negatively correlated with neutrophils, activated mast cells, and macrophages; SLC25A34 was positively correlated with M2 macrophages, CD8+ T cells, and naive B cells and negatively correlated with activated mast cells, neutrophils, and naive CD4+ T cells; ACAT1 was positively correlated with activated NK cells, memory‐activated CD4+ T cells, resting dendritic cells, CD8+ T cells, and eosinophils and negatively correlated with Tregs and M0 macrophages; PDK4 was positively correlated with naive B cells, M2 macrophages, plasma cells, and resting mast cells and negatively correlated with activated mast cells and neutrophils (Figure 4c, File S3).

Quantitative statistical analysis of CIBERSORT results revealed that plasma cells and macrophages accounted for the largest proportion (Figure 5a,b). In CRC tissues, the proportion of lymphoid cells, such as B cells and T cells, decreased, while the proportion of macrophages increased (Figure 5c).

Figure 5Immunoinfiltration analysis and immunotarget correlation analysis. (a) Proportion of CIBERSORT results in a band plot. (b) Boxplot of CIBERSORT results. (c) Boxplot of CIBERSORT results grouping. (d) Boxplot of xCELL results grouping. (e) The xCELL algorithm calculates the immune infiltration of the diagnostic model genes in CRC, with the correlation between immune cells in the upper right corner and the correlation between each type of immune cell in the lower left corner. (f) Correlation heat map of eight key genes with immune targets ( ^∗^ *p* < 0.05;  ^∗∗^ *p* < 0.01;  ^∗∗∗^ *p* < 0.001).

**Figure:**
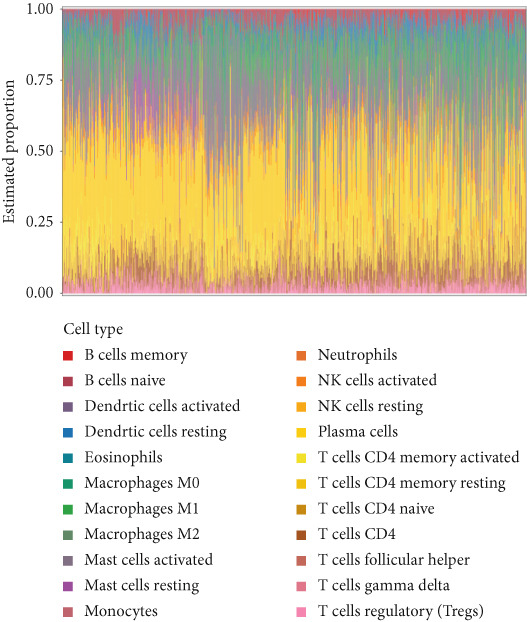
(a)

**Figure:**
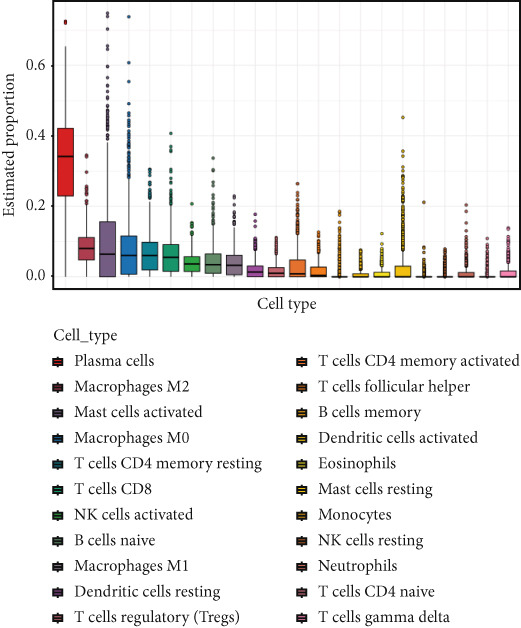
(b)

**Figure:**
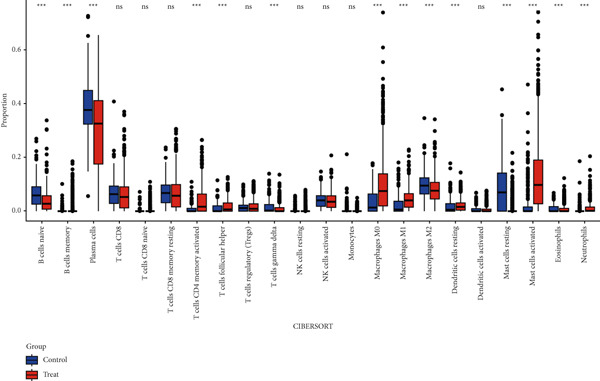
(c)

**Figure:**
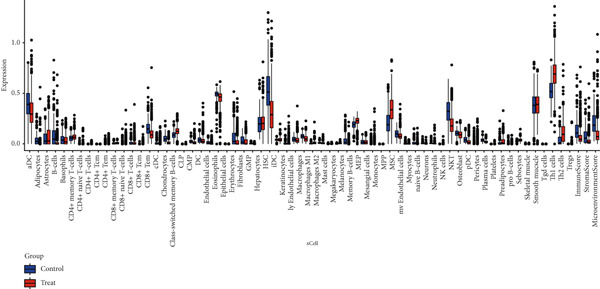
(d)

**Figure:**
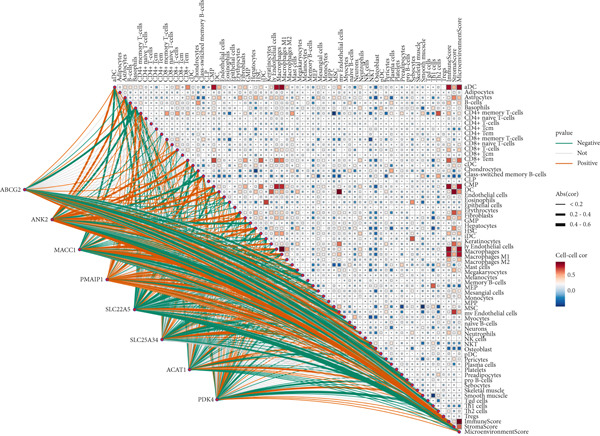
(e)

**Figure:**
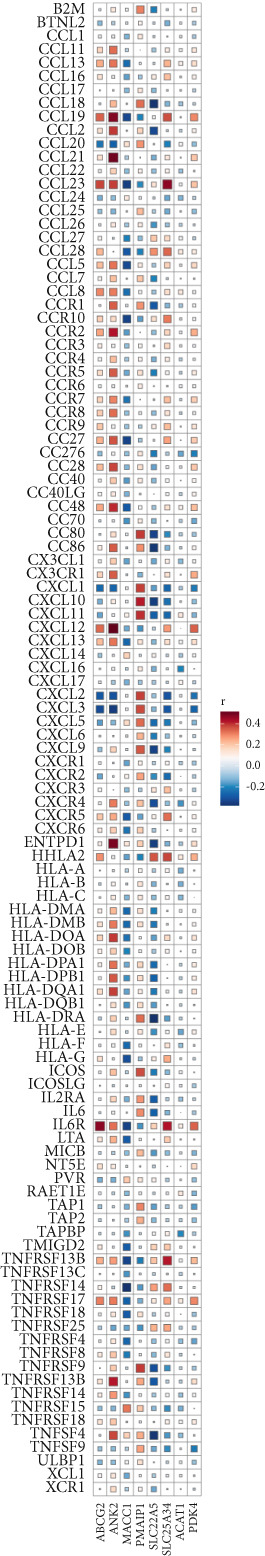
(f)

We then used the xCell algorithm for further analysis of the immune microenvironment, which showed similar trends to the CIBERSORT results, with a decrease in lymphoid cell proportions and an increase in macrophage proportions (Figure 5d). Among the eight key genes, ANK2 and PMAIP1 exhibited positive correlations with various immune cells, while SLC22A5 and SLC25A34 exhibited negative correlations (Figure 5e).

In the CRC tumor group, we analyzed the correlations between these eight key genes and 122 immune‐related targets, including immunostimulators, chemokines, receptors, and MHC sites (File S4). The results showed that ANK2 and PMAIP1 were positively correlated with multiple immune targets, while SLC22A5 and SLC25A34 were negatively correlated with several immune targets (Figure 5f).

Based on the above analyses, it is evident that the genes in the diagnostic model likely exhibit synergistic effects in CRC. They play crucial roles in tumor drug resistance, metabolic reprogramming, apoptosis regulation, and signal pathway modulation and are closely linked to immune cell infiltration in the TME.

We conducted two aspects of analysis using the TCGA‐CRC cohort: First, we counted the expression differences of the eight core genes in different tumor stages and tumor grades (Figures S9 and S10). The results showed that no obvious expression changes of the eight genes were found in the N stage and M stage via boxplots. Second, we performed multivariate Cox regression analysis using the eight core genes. The results showed that in the multivariate Cox analysis (Figure S8C), the expression levels of three core genes were significantly correlated with patients′ overall survival: ANK2 (HR = 0.93, 95% CI: 0.88–0.97, *p* = 0.0492) as a protective prognostic factor and PMAIP1 (HR = 1.03, 95% CI: 1.01–1.05, *p* < 0.001) and MACC1 (HR = 1.39, 95% CI: 1.10–1.75, *p* = 0.006) as poor prognostic factors.

### 3.5. Expression Characteristics of the Diagnostic Model at the Single‐Cell Level

We performed quality control on the single‐cell data using the Seurat R package, removing doublets and dead cells (Figure S5A,B). As a result, we retained 10,499 normal cells and 54,204 tumor cells. Highly variable genes were identified for downstream analysis (Figure S5C). PCA was then conducted using the jackstraw method to assess the significance of each principal component (PC). The Top 15 PCs with the lowest *p* values were selected for further analysis, and batch effects were removed using the RunHarmony algorithm (Figure S5D).

Based on the processed data, t‐SNE was used to cluster cells into 24 distinct clusters (Figure 6a). Marker genes for each cluster were identified (Figure 6b), and cell types were annotated by integrating marker gene expression with the “singleR” package. The annotated cell types included the following: B cells (10,784), CMPs (2696), DCs (2696), endothelial cells (2696), epithelial cells (13,480), monocytes (8088), neutrophils (2696), NK cells (2696), smooth muscle cells (2696), T cells (13,479), and tissue stem cells (2696) (Figure 6c). Marker‐based verification was performed again for the annotated clusters (Figure 6d).

Figure 6Dimensionality reduction and cell cluster identification of single‐cell t‐SNE. (a) t‐SNE was used to cluster cells into 24 distinct clusters. (b) Marker genes for each cluster were identified. (c) Cell types were annotated by integrating marker gene expression with the “singleR” package. (d) Marker‐based verification was performed again for the annotated clusters.

**Figure:**
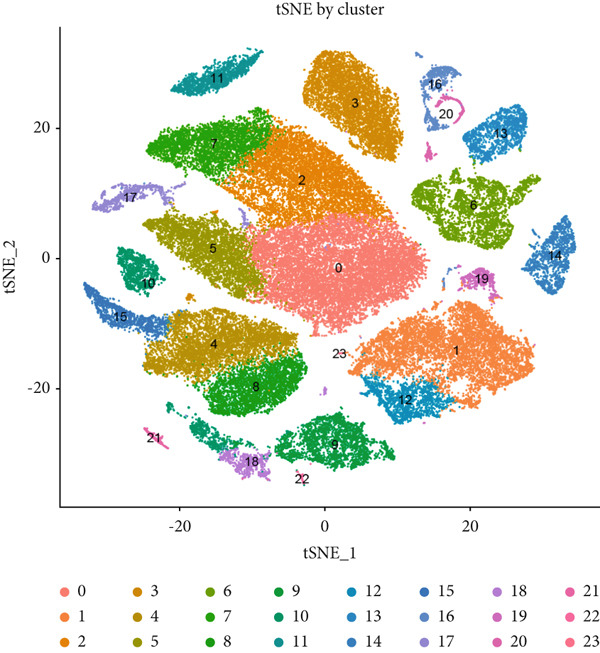
(a)

**Figure:**
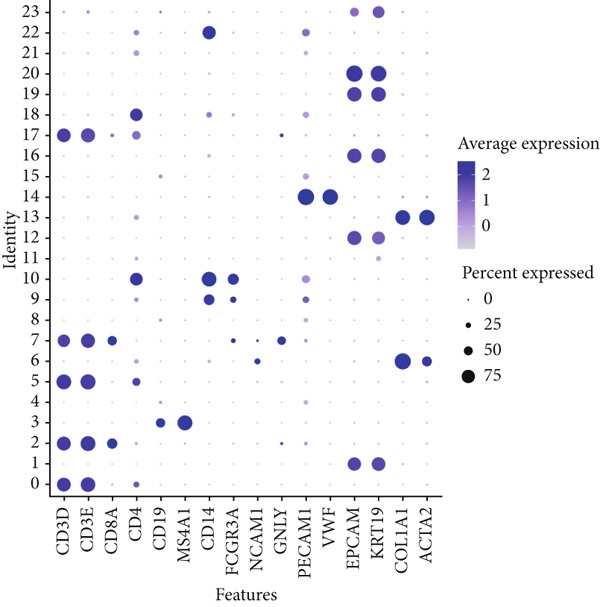
(b)

**Figure:**
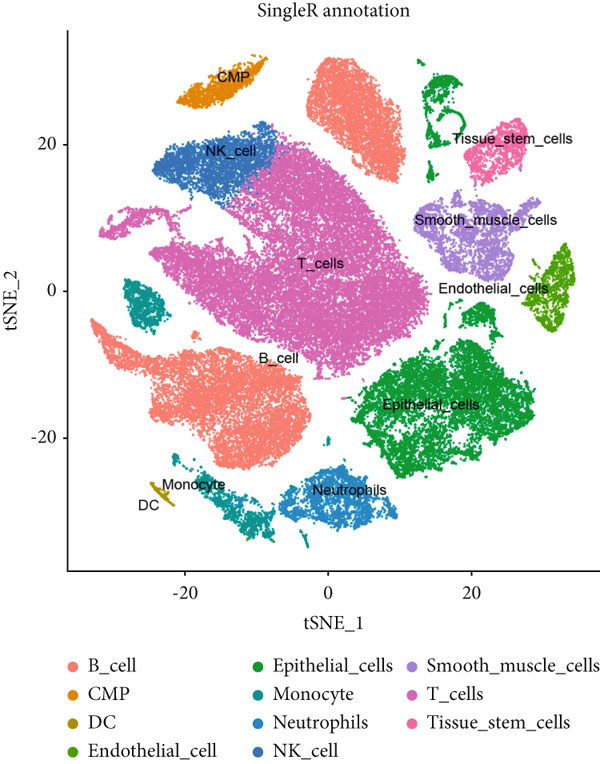
(c)

**Figure:**
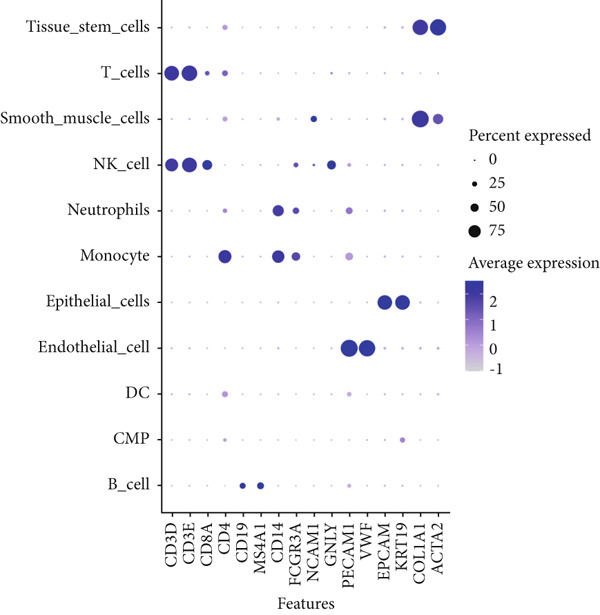
(d)

We further visualized the expression patterns of eight core genes using t‐SNE. ANK2 was mainly expressed in smooth muscle cells, MACC1 was predominantly expressed in epithelial cells, and PMAIP1 was primarily expressed in T and B cells (Figure 7b).

Figure 7Dimensionality reduction and cell cluster identification of single‐cell t‐SNE. (a) Bubble plots of eight core genes from different sample sources and cell types in single‐cell data. (b) Feature plots of eight core genes in single‐cell data. (c) Violin plots of eight core genes from different sample sources and cell types in single‐cell data ( ^∗^ *p* < 0.05;  ^∗∗^ *p* < 0.01;  ^∗∗∗^ *p* < 0.001).

**Figure:**
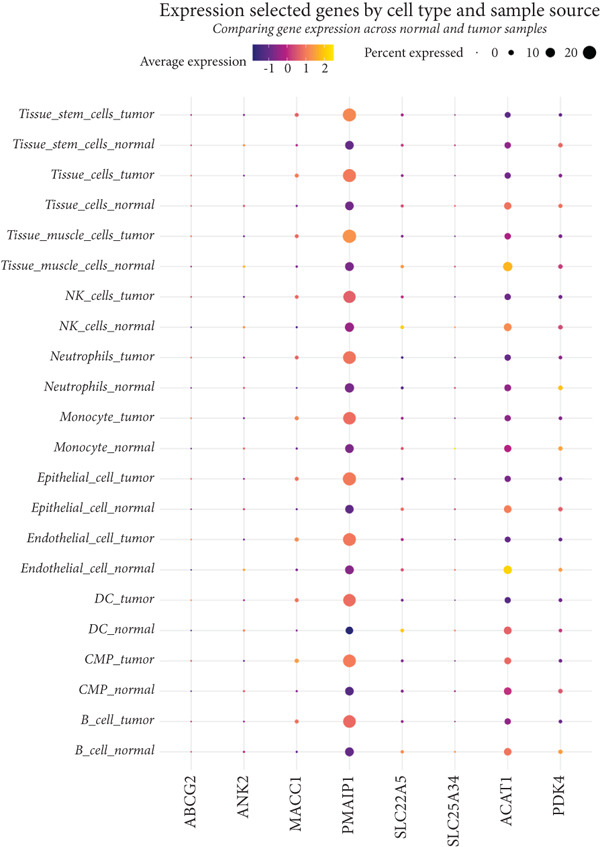
(a)

**Figure:**
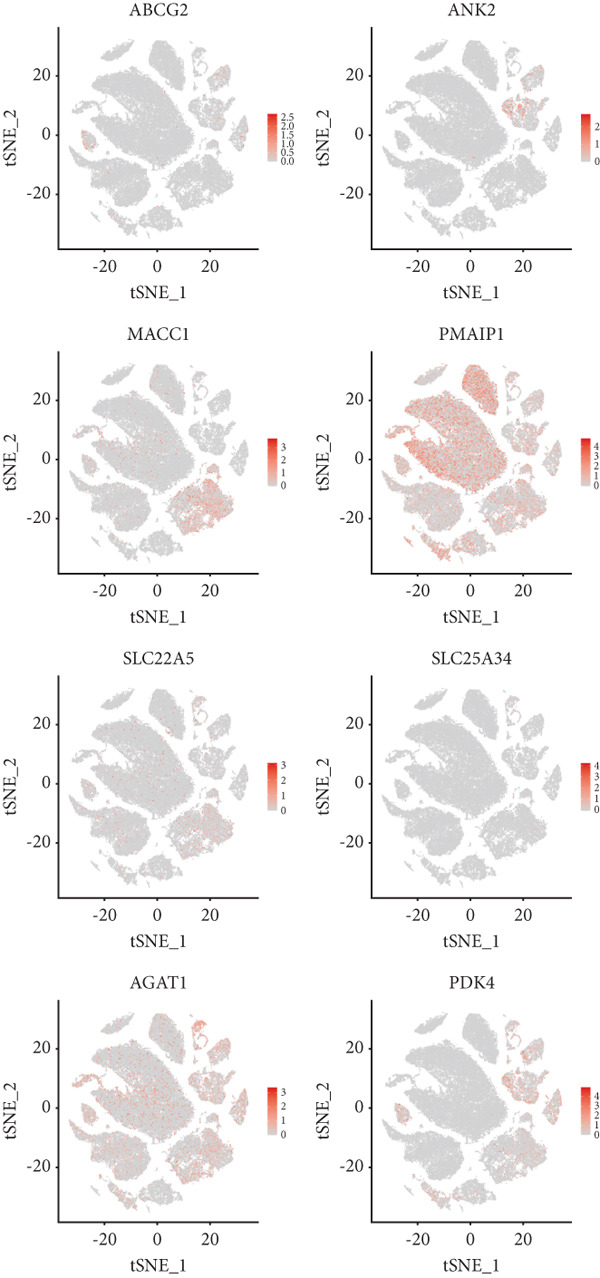
(b)

**Figure:**
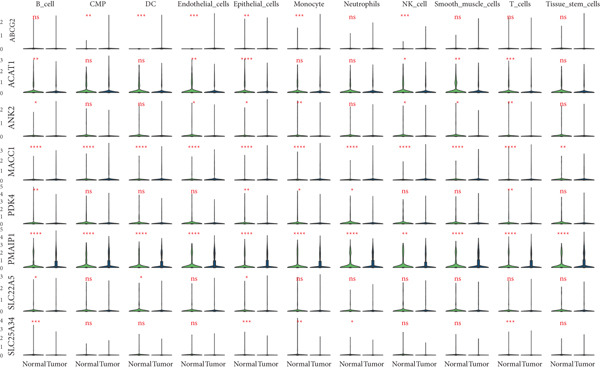
(c)

A bubble plot was used to display the expression profiles of the diagnostic model genes across different cell types (Figure 7a). Notably, ANK2 showed high expression in smooth muscle cells of normal tissues; MACC1 and PMAIP1 were significantly upregulated in various tumor cell types, particularly epithelial cells and monocytes. PDK4 and ACAT1 also showed generally high expression in tumor samples. SLC25A34 exhibited significant differential expression in epithelial cells and T cells, suggesting its potential role in regulating energy metabolism in these cells. ABCG2 was upregulated in monocytes and endothelial cells, which may be related to drug efflux or resistance mechanisms (Figure 7c).

### 3.6. Apoptosis Scoring in Single‐Cell Data

We utilized AUCell to score the apoptosis gene set (File S6) across single‐cell samples, presenting the results separately for all samples, normal samples, and cancer samples (Figure 8a). A comparison of different cell types between normal and cancer samples revealed that apoptosis scores were significantly higher across all cell types in cancer samples compared to normal ones (Figure 8b). Furthermore, in cancer samples, ANK2 exhibited an opposite trend to the apoptosis score (Figure 8c).

Figure 8Single‐cell data apoptosis dataset score. (a) Feature plots of apoptosis dataset score genes in single‐cell data. (b) Apoptosis score grouping boxplots for different cell types between normal and cancer samples. (c) The boxplot of the immune score and the line chart of the expression levels of the eight core genes: The *y*‐axis on the left is the immune score value, and the *y*‐axis on the right is the gene expression value ( ^∗^ *p* < 0.05;  ^∗∗^ *p* < 0.01;  ^∗∗∗^ *p* < 0.001).

**Figure:**
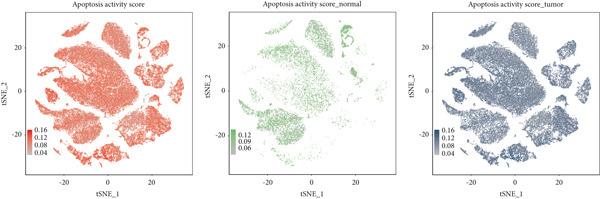
(a)

**Figure:**
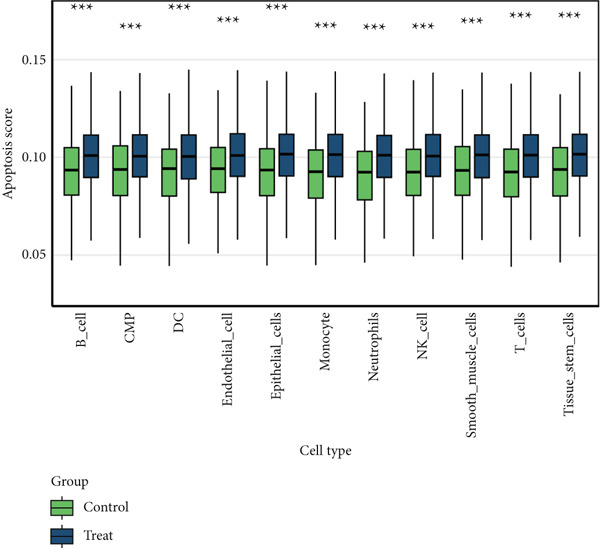
(b)

**Figure:**
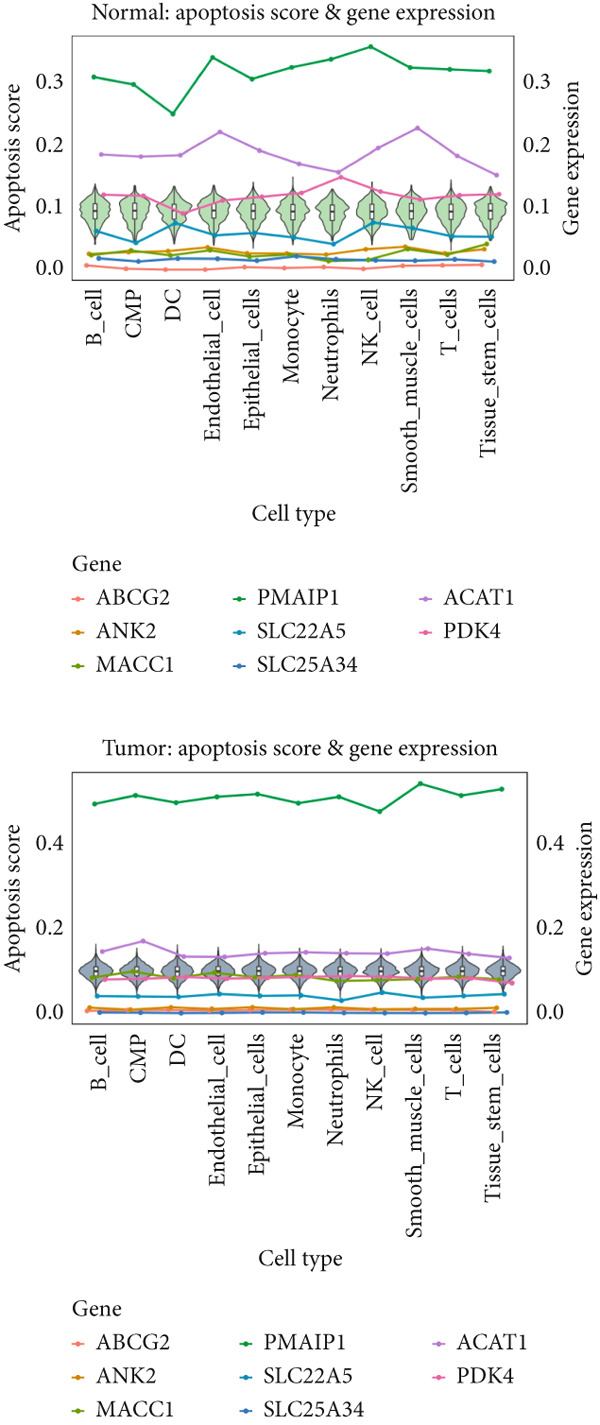
(c)

### 3.7. Validation of Low Expression of ANK2 in CRC

In the process of investigating the reliability and mechanisms of the diagnostic model genes, we focused on existing studies on these genes. Except for ANK2, the other seven genes have been extensively studied in CRC. Among them, ABCG2 is an important member of the ABC transporter superfamily. Sałagacka‐Kubiak et al. confirmed its significantly low expression in CRC, supporting its potential as a reliable diagnostic marker for CRC. MACC1 has been well‐studied in CRC, with research showing that the MACC1/HGF/c‐MET axis enhances the proliferation and metastasis of colon cancer. Additionally, calcium‐binding protein S100P has been identified as a new target gene of MACC1, which drives CRC metastasis. PMAIP1, also known as NOXA, promotes apoptosis, but PRDX1 can suppress its apoptotic effect by enhancing UBE2F‐CUL5‐mediated NOXA degradation. Zou and colleagues confirmed that rs27437, located in the SLC22A5 gene, is significantly associated with CRC risk in the Chinese population. It is suggested that rs27437 might reduce SLC22A5 expression by affecting transcription factor binding, thereby increasing CRC risk. Li et al.′s research showed that the E3 ubiquitin ligase MYLIP mediates the NKRF/SLC25A34 axis to inhibit the malignant progression of CRC. Liu et al. demonstrated that ACAT1 is downregulated in CRC tissues and its expression correlates with tumor staging. PDK4 low expression was also verified in a study on the impact of exosome‐related genes (ERG) on CRC diagnosis.

However, there is currently no direct and specific evidence regarding the low expression of ANK2 in CRC and its mechanisms. Therefore, to confirm the role of ANK2 in CRC development, we first retrieved the immunohistochemical results of ANK2 from the HPA database to evaluate its expression in CRC tissues. The results showed that the ANK2 protein was downregulated in CRC tissues (Figure 9a). We then used qRT‐PCR and western blotting to measure the mRNA and protein expression levels of ANK2 in five CRC cell lines. The results indicated that, compared to NCM460, ANK2 expression was significantly lower in four CRC cell lines (Figure 9b), consistent with the protein levels observed in western blotting (Figure 9c).

Figure 9ANK2 is low expressed in CRC. (a) Immunohistochemistry showed that ANK2 was lowly expressed in CRC. (b) Relative mRNA expression levels of ANK2 in CRC cell lines. (c) Protein expression levels of ANK2 in CRC cell lines ( ^∗^ *p* < 0.05;  ^∗∗^ *p* < 0.01;  ^∗∗∗^ *p* < 0.001).

**Figure:**
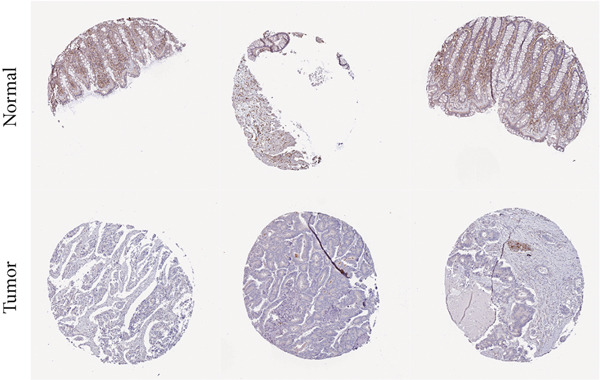
(a)

**Figure:**
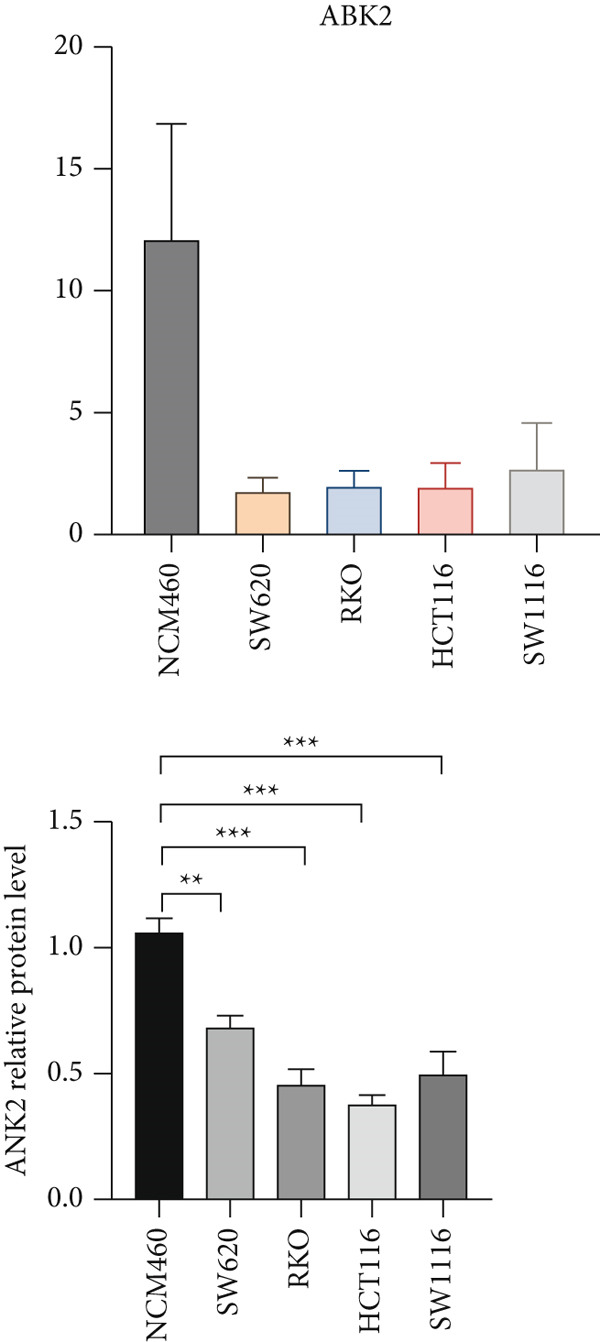
(b)

**Figure:**
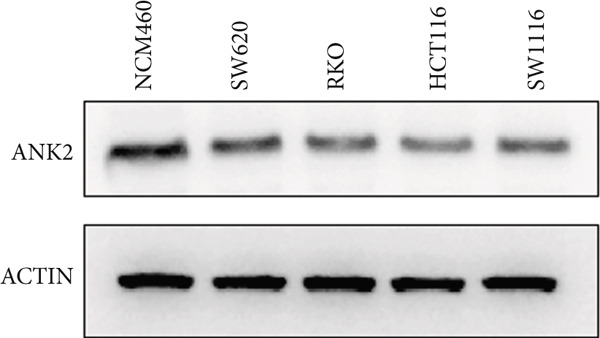
(c)

### 3.8. Promotion of Angiogenesis and Inhibition of Apoptosis by Low Expression of ANK2

Based on previous gene interaction analysis, we found that ANK2 exhibits coexpression relationships with cytoskeleton‐related genes. Subsequent single‐cell level expression analysis further revealed a significant correlation between ANK2 and smooth muscle cells, suggesting that ANK2 may be closely associated with cell apoptosis and TME angiogenesis in CRC. To validate these hypotheses, we conducted the following experiments.

First, we performed TUNEL assays on LoVo cells (Figure 10a). The intensity of green fluorescence reflects the degree of DNA fragmentation, a key hallmark of apoptosis. The ANK2‐OE group exhibited significantly stronger green fluorescence than the ANK2‐NK group, indicating a higher level of nucleosomal DNA fragmentation and suggesting that ANK2 overexpression effectively promotes apoptosis. Mechanistically, ANK2 may activate intrinsic apoptotic signaling pathways, enhancing the expression of proapoptotic factors and thereby accelerating cell death. In contrast, the ANK2‐KD group displayed markedly weaker fluorescence compared to the ANK2‐NK group, indicating reduced DNA fragmentation and suppressed apoptosis. The loss of ANK2 may impair apoptotic signaling, decreasing cellular sensitivity to apoptosis‐inducing stimuli and prolonging cell survival. These findings underscore the critical role of ANK2 in regulating cell survival and apoptosis. In summary, ANK2 overexpression significantly enhances LoVo cell apoptosis, while its knockdown inhibits apoptotic processes, providing novel experimental evidence for ANK2′s role in apoptosis regulation in both physiological and pathological contexts.

Figure 10Hypoexpression of ANK2 promotes angiogenesis, as well as inhibition of apoptosis. (a) The intensity of green fluorescence reflected the degree of intracellular DNA fragmentation, and the intensity of green fluorescence in the ANK2‐OE group was significantly higher than that in the ANK2‐NK group, indicating that the nucleosomal genomic DNA fragmentation was higher in the cells in this group, while the opposite was true in the ANK2‐KD group. (b) The number of tubular structures of HUVECs in the ANK2‐OE group was significantly higher than that in the ANK2‐NK group, while the number of tubular structures in the ANK2‐KD group was the opposite. Repeat each set three times  ^∗∗∗^ *p* < 0.001.

**Figure:**
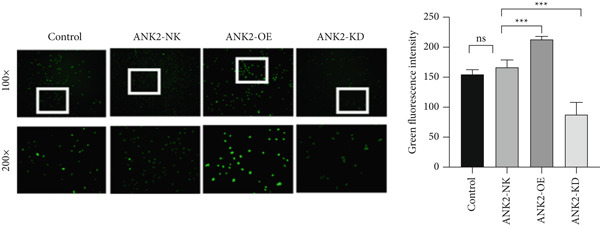
(a)

**Figure:**
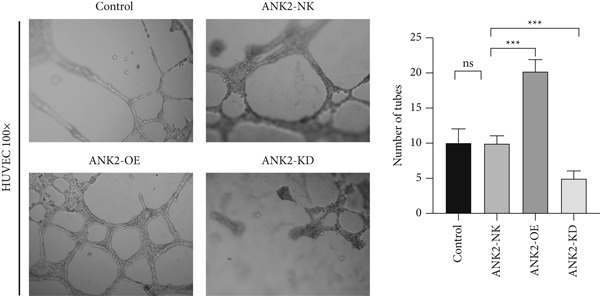
(b)

Next, we conducted tube formation assays using HUVEC cells cultured in conditioned medium (Figure 10b). Under identical culture durations, the ANK2‐OE group formed substantially more tubular structures than the ANK2‐NK control, whereas the ANK2‐KD group showed significantly fewer tubes. Moreover, the vascular networks in the ANK2‐KD group appeared simpler, with defective branching and discontinuous lumens. These results were validated in three independent experiments and demonstrated statistical significance. These findings provide experimental support for the pivotal role of ANK2 in vascular remodeling within the CRC TME, suggesting that ANK2 could serve as a potential therapeutic target for angiogenesis modulation.

## 4. Discussion

CRC remains a significant public health burden due to its high incidence and mortality rates. Although various screening and treatment strategies have been implemented clinically, challenges such as low early detection rates, limited therapeutic efficacy, and poor prognosis persist. Notably, the substantial biological heterogeneity among patients results in varied therapeutic responses, further hindering treatment success. Therefore, identifying early diagnostic biomarkers with high sensitivity and specificity is one of the critical goals in both basic and clinical CRC research.

In this study, we systematically analyzed gene expression differences between CRC and normal tissues using data from the TCGA and multiple GEO datasets. Our findings revealed widespread dysregulation of gene expression in CRC. GO and KEGG enrichment analyses indicated that these DEGs are involved in key BPs and pathways, including cell cycle regulation, metabolism, immune microenvironment modulation, tumor metastasis, protein processing in the endoplasmic reticulum, DNA replication, HTLV‐1 infection, apoptosis, p53 signaling, and carbon metabolism. GSEA results further suggested that disruptions in pathways from DNA replication initiation and cell cycle deregulation to necroptosis and sustained Wnt signaling play central roles in CRC tumorigenesis and progression. In recent years, mitochondria have been increasingly recognized for their vital roles in cellular homeostasis. As the cell′s “powerhouse,” mitochondria are central not only to energy metabolism but also to oxidative stress responses, apoptosis regulation, and immune modulation, all of which are closely linked to cancer development. Consequently, mitochondrial function and related genes may offer promising avenues for early CRC diagnosis and therapeutic targeting.

Building on this premise, we integrated MRGs with DEGs and employed two machine learning algorithms, LASSO and SVM‐RFE, to develop a diagnostic model comprising eight key genes: ABCG2, ANK2, MACC1, PMAIP1, SLC22A5, SLC25A34, ACAT1, and PDK4. These genes consistently showed significant dysregulation in CRC across public datasets, suggesting their biological relevance in tumorigenesis. We subsequently examined recent research advances on each of these genes.

For instance, ABCG2 is a vital component of the ABC transporter superfamily. Studies by Sałagacka‐Kubiak et al. have confirmed its significantly reduced expression in CRC, supporting its potential as an early diagnostic biomarker [10]. Regarding MACC1, research has shown that under the regulation of the MACC1/HGF/c‐MET axis, it promotes the proliferation and metastasis of CRC cells. Additional evidence suggests that MACC1 can drive tumor metastasis via the calcium‐binding protein S100P [11, 12]. PMAIP1 encodes NOXA, a proapoptotic BH3‐only protein that is generally overexpressed in CRC. However, its rapid degradation through ubiquitination leads to a shortened half‐life, which explains its limited functional impact despite high expression [13]. PRDX1 shows a negative correlation with NOXA stability, promoting its ubiquitination and degradation, thereby helping CRC cells evade apoptosis. SLC22A5, a member of the solute carrier (SLC) family, encodes the high‐affinity carnitine transporter OCTN2, which plays a crucial role in shuttling l‐carnitine into cells and supporting fatty acid *β*‐oxidation [14]. Under physiological conditions, SLC22A5 is highly expressed in the intestine, liver, and kidney, maintaining metabolic homeostasis. Zou et al. confirmed its downregulation in CRC and proposed that the rs27437 variant may affect upstream transcription factor binding, leading to decreased gene expression and increased cancer risk [15]. Furthermore, Li et al. found that in inflammatory bowel disease (IBD), reduced OCTN2 expression impaired l‐carnitine uptake by intestinal epithelial cells, disrupting tissue repair, aggravating inflammation, and contributing to poor prognosis underscoring its diagnostic relevance in early stage CRC [16]. SLC25A34, a member of the mitochondrial inner membrane SLC25 transporter family, mediates the transmembrane transport of metabolites such as amino acids and nucleotides. Studies have shown that its expression is significantly decreased in CRC tissues and cell lines, correlating with increased tumor aggressiveness, metastasis, and poor prognosis [17]. ACAT1 is a key enzyme in ketone body and lipid metabolism. Research by Liu et al. revealed that ACAT1 is downregulated in CRC tissues and is associated with tumor stage, highlighting its potential value in CRC diagnosis [18]. PDK4, a critical regulator of energy metabolism, was found to be significantly downregulated in CRC in a study exploring the role of ERGs in CRC diagnostics, suggesting its involvement in metabolic reprogramming during cancer progression [19]. ANK2 encodes a member of the ankyrin family of proteins involved in cytoskeletal organization, membrane stability, and signal transduction. However, current studies on its role in CRC remain limited, and its biological significance requires further investigation.

We thoroughly evaluated the diagnostic model through multiple approaches. ROC analysis demonstrated excellent diagnostic performance (AUC > 0.9) for all eight genes. GeneMANIA revealed tight interactions via coexpression and shared structural domains among these genes. CIBERSORT and xCell analyses showed that these genes are significantly associated with various immune cell subtypes, suggesting a potential role in modulating the TME.

Single‐cell transcriptomic analysis further clarified gene expression specificity across different cell populations. MACC1 was highly expressed in epithelial cells, suggesting a role in epithelial proliferation and malignant transformation. PMAIP1 was upregulated in T and B cells, implicating it in angiogenesis and immune evasion. ANK2 showed high expression in smooth muscle cells of normal tissue but was significantly downregulated in CRC. Moreover, ANK2 expression negatively correlated with apoptosis scores across cell types; higher apoptosis scores corresponded to lower ANK2 expression, suggesting its involvement in structural stability and vascular regulation.

To validate ANK2′s function, we performed qPCR, western blot, TUNEL, and tube formation assays. ANK2 was significantly downregulated in CRC cell lines, and its suppression led to reduced apoptosis and enhanced angiogenesis, consistent with our bioinformatics predictions. These findings support ANK2′s tumor‐suppressive potential and highlight it as a promising biomarker.

Increasing evidence supports the superiority of multigene models over single biomarkers for tumor detection. Our mitochondria‐related diagnostic model, constructed from public data and machine learning algorithms, exhibits robust predictive performance, offering a reliable foundation for early CRC detection.

Nonetheless, this study has limitations. Public datasets vary in ethnic background and technical platforms, potentially introducing batch effects. Clinical utility of the model requires validation in large, multicenter prospective studies. Furthermore, although ANK2′s function was explored in vitro, its roles in animal models and clinical samples warrant further investigation to elucidate its mechanisms in tumor development and treatment response.

In conclusion, this study integrates multiomics data and experimental validation to establish a mitochondria‐related multigene diagnostic model for CRC and highlights the tumor‐suppressive role of ANK2. Future research should focus on clinical translation and in‐depth exploration of mitochondrial pathways and their interactions with the TME to facilitate more effective strategies for early tumor detection and intervention.

## 5. Conclusions

In this study, we developed a diagnostic model for CRC based on mitochondria‐related DEGs, using both LASSO and SVM machine learning algorithms. Within this model, ABCG2, ANK2, SLC22A5, SLC25A34, ACAT1, and PDK4 were found to be downregulated in CRC, whereas MACC1 and PMAIP1 were upregulated.

We applied multiple bioinformatics approaches to explore the potential synergistic interactions among these diagnostic genes in CRC. The results revealed a strong correlation between the model genes and immune cell infiltration in CRC. Moreover, we analyzed the expression patterns of these genes at the single‐cell level, identifying their associations with various TME stromal and immune cell types.

Furthermore, we selected ANK2 from the diagnostic model for additional experimental validation. Consistent with our bioinformatics predictions, ANK2 was significantly downregulated in CRC cells and tissues. Its low expression was associated with enhanced angiogenesis in the TME and suppressed apoptosis in tumor cells.

In conclusion, the MRG‐based diagnostic model established in this study may provide effective novel biomarkers for the early detection of CRC and offer a reliable basis for more accurate diagnosis in CRC patients.

## Conflicts of Interest

The authors declare no conflicts of interest.

## Author Contributions

X.D.: validation, visualization, writing—original draft, and writing—review and editing; H.W.: validation and writing—review and editing; J.Y.: validation and writing—original draft; H.S.: visualization and writing—review and editing; J.G.: supervision and writing—review and editing; X.W.: conceptualization, funding acquisition, supervision, and writing—review and editing; X.Z.: conceptualization, resources, supervision, and writing—review and editing. X.D., H.W., and J.Y. contributed equally to this work and share the first authorship.

## Funding

This study was supported by the Postgraduate Research & Practice Innovation Program of Jiangsu Province (KYCX24_3667).

## Supporting information


**Supporting Information** Additional supporting information can be found online in the Supporting Information section. Figure S1: GSEA enrichment results. Table S2: Mitochondrial gene set. Figure S3: Correlation analysis between immune infiltration results and eight core genes. Table S4: One hundred twenty‐two immune target gene sets. Figure S5: Single‐cell data quality control. Table S6: Apoptosis gene set. Figure S7: PCA plot. Figure S8: GO analysis, KEGG analysis, and multivariate Cox regression. Figure S9: N stage correlation analysis. Figure S10: M stage correlation analysis.

## Data Availability

The corresponding authors can supply all data generated or analyzed in this study/supporting information upon a justified request.
