# Supplementary material for: Construction of a Mitochondria‐Related Gene Diagnostic Model Based on Integrated Multiomics Data and Functional Validation of ANK2 as a Key Regulator in Colorectal Cancer
Source: Int J Genomics. 2026 Jan 31;2026:9306920. doi: 10.1155/ijog/9306920 (PMC12860396; doi:10.1155/ijog/9306920)
Supplement: Supplementary file 1 — Supporting Information Additional supporting information can be found online in the Supporting Information section. Figure S1: GSEA enrichment results. Table S2: Mitochondrial gene set. Figure S3: Correlation analysis between immune infiltration results and eight core genes. Table S4: One hundred twenty‐two immune target gene sets. Figure S5: Single‐cell data quality control. Table S6: Apoptosis gene set. Figure S7: PCA plot. Figure S8: GO analysis, KEGG analysis, and multivariate Cox regression. Figure S9: N stage correlation analysis. Figure S10: M stage correlation analysis. [file IJOG-2026-9306920-s001.docx]

**Supplementary Material**

**
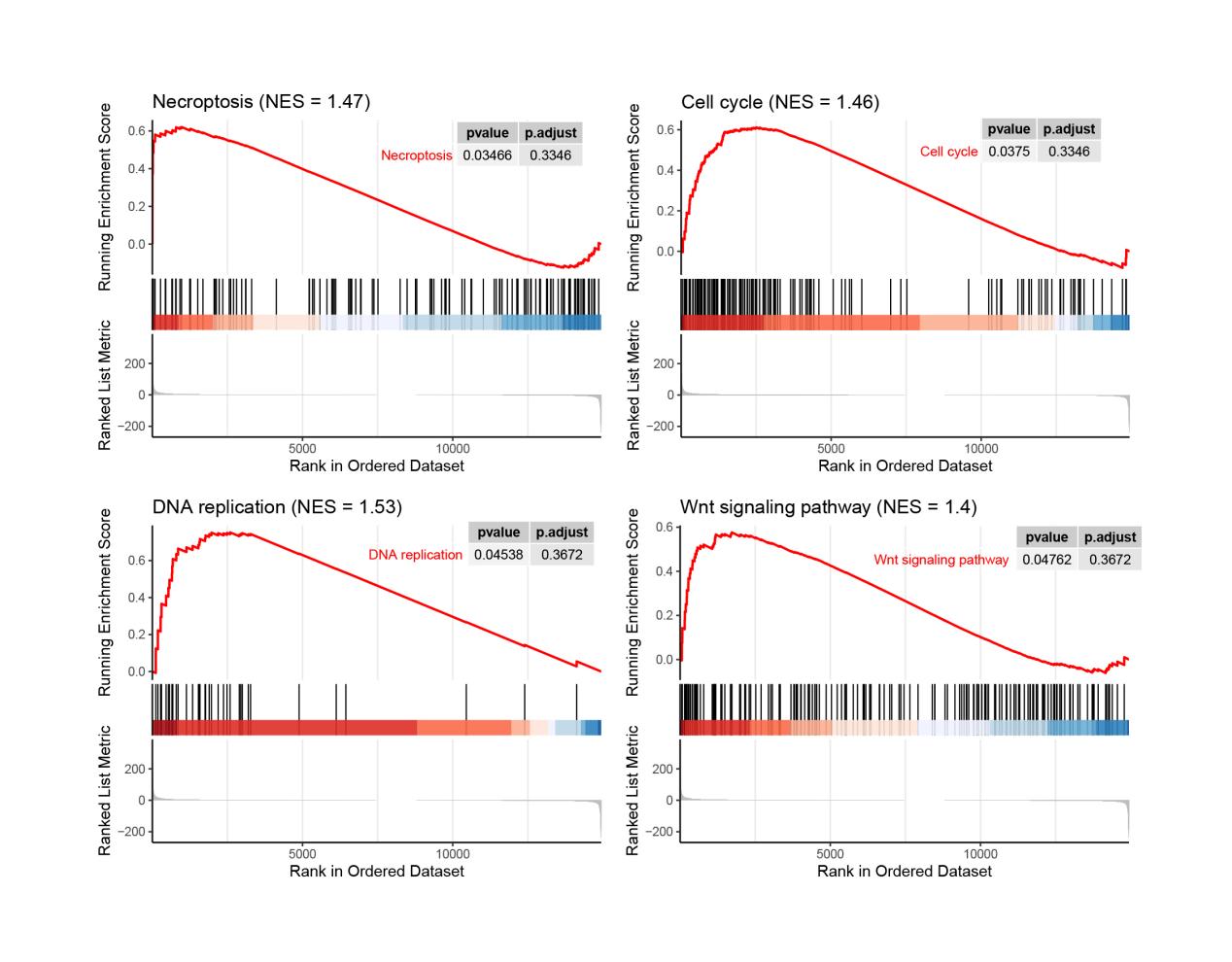
**

**Figure S1 GSEA enrichment results**

ABCF2

ACBD6

ACBD7

ACE

ACE2

ACOT1

ACOT12

ADORA2A

AGTR1

AGTR2

AKT2

ALOX12

ARL6IP5

CAMK4

CARM1

CASP7

CAV3

CCL27

CGAS

CHD9

CHKB

CREBBP

CRTC1

CRTC2

CRTC3

CTSL

DDX58

ELAVL1

G6PD

GABPB1-IT1

GOT1

HDAC3

HELZ2

HMGA1

HSD17B12

IFIH1

IL1B

IL6

IRF7

MAPK11

MDH1

MED1

MEF2C

MEF2D

MID1IP1

MIR181B1

MIR210

MT-TC

MT-TI

MT-TK

MT-TP

MT-TT

MT-TV

MYEF2

NAMPT

NCOA1

NCOA2

NCOA6

NCOR1

NFKB2

NMNAT2

NOX1

NR1D1

OPRD1

PCTP

PERM1

PIP5KL1

PM20D1

PPARA

PPP2R3C

PRKAA2

PRKAB1

PRKAB2

PRKAG1

PRKAG2

PRKAG3

RB1

RELA

RNF122

SLC2A1

SLC2A4

SMARCD3

SNORD138

SP1

STARD3

TBL1X

TBL1XR1

TCL1A

TGS1

THRSP

TLR3

TLR7

TMPRSS2

TRAF6

USP46

AARS1

ABCE1

ABCG1

ABCG2

ABHD4

ABHD5

ABHD6

ABHD8

ABL1

ABLIM3

ACAA2

ACAT2

ACBD3

ACO1

ACO2

ACOD1

ACOT11

ACOT13

ACSBG2

ACSL3

ACSL4

ACSL5

ACSL6

ACSM1

ACSM2A

ACSM2B

ACSM4

ACSM5

ACSM6

ACSS1

ACSS2

ACTR10

ADAM28

ADAP2

ADH5

ADPRS

ADSS2

AFG3L2

AGBL4

AGPS

AGTPBP1

AGXT2

AIFM3

AIM2

AIP

AK2

AK4

AK9

AKAP8

AKR1B10

AKR1B15

AKT1

AKT3

ALAS2

ALB

ALDH18A1

ALDH1B1

ALDH5A1

ALDH6A1

ALKBH3

AMACR

AMBRA1

AMMECR1

ANK2

ANKRD37

ANTKMT

ANXA1

ANXA6

AP3B1

APEX1

APEX2

APH1A

AQP8

ARAF

ARG1

ARGLU1

ARID4B

ARIH2

ARL2BP

ARMC1

ARMC10

ARMS2

ARRB2

ARSB

ASAH2

ASB9

ASS1

ATAD3A

ATAD3B

ATAD3C

ATCAY

ATF2

ATG12

ATG13

ATG14

ATG2A

ATG2B

ATG3

ATG4D

ATG5

ATG7

ATG9A

ATG9B

ATP13A2

ATP2A1

ATP5F1B

ATP5F1C

ATP5F1E

ATP5F1EP2

ATP5MC3

ATP5MGL

ATP5PO

ATP7A

ATP7B

ATPAF1

ATXN3

AURKAIP1

AVP

AZIN2

BAG3

BAG4

BAG5

BAP1

BBC3

BBOX1

BCAP31

BCAT1

BCKDK

BCL2

BCL2A1

BCL2L1

BCLAF3

BDNF

BECN1

BHLHA15

BLID

BLOC1S2

BMF

BNIP1

BRAT1

BRD8

BRI3BP

BRINP3

BSG

BTD

C10orf67

C10orf88

C11orf65

C12orf73

C14orf119

C15orf62

C19orf12

C1orf43

C9orf72

CABS1

CALM1

CALM3

CAMK2A

CAMKK2

CAMKMT

CAPN1

CAPN10

CAPN2

CAPRIN2

CARD19

CARS2

CASP1

CASP14

CASP2

CASP4

CASP8AP2

CASQ1

CAT

CAV2

CAVIN1

CCAR2

CCDC51

CCDC58

CCK

CCM2

CCN6

CCNB1

CCR7

CDC25C

CDC37

CDK1

CDK5

CDK5RAP1

CDS2

CEBPA

CEBPZOS

CEND1

CEP89

CERT1

CFAP410

CFAP91

CHCHD1

CHCHD10

CHCHD6

CHCHD7

CHPF

CIAPIN1

CIBAR1

CIDEA

CIDEB

CISD2

CKMT1B

CLIC1

CLIC4

CLN8

CLPX

CLU

CLUH

CMC1

CNP

CNR1

COA3

COA4

COA5

COL7A1

COQ10A

COQ10B

COQ3

COQ4

COQ5

COQ8B

COQ9

COX11

COX17

COX18

COX19

COX20

COX4I1

COX4I2

COX7A2L

COX7A2P2

COX7C

CPNE3

CPS1

CPT1B

CPT1C

CREB1

CREB3L4

CREBZF

CRYAB

CSNK2A2

CTNS

CTSK

CTTN

CWC15

CXADR

CYB5A

CYB5R1

CYBA

CYBB

CYP1A1

CYP1B1

CYP27A1

CYP27C1

CYP2D6

CYP2D7

CYP2E1

CYP2U1

CYRIB

DAO

DAOA

DBT

DCAF8

DCK

DCN

DCPS

DCTPP1

DDAH2

DDHD1

DDHD2

DDIT3

DDIT4

DDX1

DDX21

DDX6

DECR1

DEGS1

DEPP1

DGAT2

DHFR

DHFR2

DHFRP1

DHRS13

DHX32

DHX36

DIMT1

DIP2A

DISC1

DLGAP5

DNAJA1

DNAJA3

DNAJC11

DNAJC15

DNAJC19

DNAJC5

DND1

DNM1

DNM2

DNM3

DNMT1

DOK7

DUSP18

DUSP21

DYNLL1

DYNLT1

E2F1

EARS2

ECHDC2

ECHS1

EFHD1

EHHADH

ELK1

ELK3

ENOSF1

ENY2

EP300

EPAS1

EPHA4

ERAL1

ERBB4

ERCC6L2

ERN1

ESR2

ESRRA

ETFB

ETFBKMT

ETFDH

ETNPPL

EXD2

EYA2

FADS1

FAM110B

FAM124B

FAM162A

FAM72A

FANCG

FASTKD3

FASTKD5

FATE1

FBXL4

FBXO7

FBXW7

FDX2

FECH

FEN1

FEZ1

FGR

FH

FHIT

FIBP

FKBP4

FLCN

FLVCR1

FNIP1

FOXK2

FOXO1

FOXO3

FPGS

FTMT

FUNDC1

FUNDC2

FUS

FXN

FYN

FZD5

FZD9

G0S2

GABARAP

GABARAPL1

GABARAPL2

GABARAPL3

GABPA

GABPB1

GADD45GIP1

GARS1

GATB

GATD3B

GATM

GBA

GCAT

GCK

GCKR

GCLM

GCSH

GDAP1

GDF5-AS1

GFM1

GGCT

GGNBP1

GHITM

GIMAP8

GIT1

GJA1

GK

GK2

GK5

GLRX2

GLRX5

GLUD2

GLUL

GLYAT

GLYATL1

GLYATL1B

GLYATL2

GLYATL3

GNE

GNPAT

GNRH1

GOLPH3

GOT2

GPAA1

GPER1

GPN1

GPS2

GRAMD4

GRPEL1

GRSF1

GSK3A

GSK3B

GSTP1

GSTZ1

GTF3C4

GTPBP10

GTPBP3

GTPBP8

GUF1

GYG1

GZMB

H6PD

HADHA

HAGH

HAP1

HARS1

HAUS3

HAX1

HCFC1

HCLS1

HDAC6

HEATR1

HEBP2

HEMK1

HGF

HIF1A

HIF3A

HIGD1A

HIGD1B

HIGD1C

HIGD2A

HIGD2B

HIP1R

HIVEP1

HJURP

HK1

HK2

HK3

HKDC1

HLCS

HMGN5

HPS4

HRK

HS1BP3

HSD3B1

HSD3B2

HSP90AA1

HSP90AB1

HSP90B2P

HSPA13

HSPA1A

HSPA1B

HSPA1L

HSPA4

HSPA5

HSPA9

HTD2

HTT

HUWE1

HYKK

IAPP

IBA57

IDE

IDH1

IDH3G

IFI27

IFI27L1

IFI27L2

IFI6

IFIT2

IFIT3

IGF1

IKBKE

ILF3

IMMP1L

IMMP2L

IMMT

INF2

IQCN

IREB2

IRF3

IRGM

ISCU

JARID2

JTB

JUN

KANK2

KAT2A

KCNJ11

KCNJ8

KDR

KIF1B

KIF28P

KIFBP

KLC2

KLK6

KMO

KRAS

KYAT1

KYAT3

KYNU

LACTB

LACTB2

LARS2

LDHAL6B

LDHD

LEPROT

LGALS3

LIG1

LIPF

LIPT1

LMAN1

LPIN1

LRPPRC

LRRC10

LRRC59

LRRK1

LRRK2

LYN

MACC1

MAFF

MALSU1

MAN2A1

MAP1B

MAP1LC3A

MAP1LC3B

MAP1LC3B2

MAP1LC3C

MAP1S

MAP2K1

MAP2K2

MAPK1

MAPK10

MAPK12

MAPK14

MAPK3

MAPK8

MAPK8IP1

MAPK9

MAPT

MARK1

MARK2

MAVS

MCCC1

MCCD1

MCEE

MCL1

MCUB

ME1

ME2

MEF2A

MGARP

MICALL2

MICOS10

MICOS13

MICU3

MIGA1

MIR144

MIR17

MIR29A

MIR29B1

MIR29C

MLLT11

MLXIP

MMAA

MMAB

MMP1

MMP2

MMP9

MOAP1

MOBP

MPC1

MPC1L

MPC2

MPG

MRM3

MRPL10

MRPL18

MRPL19

MRPL20

MRPL21

MRPL24

MRPL32

MRPL51

MRPL52

MRPL58

MRPS17

MRPS18B

MRPS28

MRPS30

MRPS34

MRPS35

MRPS36

MSRB2

MSTO1

MTCH2

MT-CO1

MT-CO2

MTCO2P12

MTERF2

MTERF3

MTERF4

MTG2

MTHFD1

MTHFD1L

MTLN

MTM1

MT-ND5

MTO1

MTOR

MTRF1L

MT-RNR1

MT-RNR2

MTRNR2L5

MT-TE

MT-TF

MT-TH

MT-TL1

MT-TL2

MT-TN

MT-TQ

MT-TS2

MT-TW

MTUS1

MTX3

MX1

MX2

MXD1

MYCBP

MYH14

MYH7

MYL10

MYO19

MYOC

MYOM2

NACC2

NAIF1

NAPG

NAXD

NAXE

NCBP1

NCSTN

NDFIP2

NDRG4

NDUFA3

NDUFA4L2

NDUFAF2

NDUFAF3

NDUFAF5

NDUFB1

NDUFB10

NDUFB11

NDUFB4

NDUFB5

NDUFC2-KCTD14

NDUFS5

NDUFS6

NDUFS8

NEB

NECTIN2

NEFL

NENF

NFKB1

NFS1

NGB

NGDN

NIPSNAP1

NIPSNAP2

NIPSNAP3A

NLRP5

NME1

NME2

NMT1

NOC3L

NOD2

NOL3

NOL6

NOL7

NOS1

NOS1AP

NOS3

NOX4

NPEPPS

NR3C1

NR4A1

NRF1

NRGN

NRP1

NT5M

NTHL1

NTSR1

NUDT1

NUDT13

OAS1

OGT

OLFM4

OPTN

OXCT1

P2RY1

P2RY12

P4HA1

PACS2

PAGE4

PAK5

PAM16

PANK2

PARG

PARL

PARP1

PARP9

PC

PCCB

PCF11

PCK1

PDCD5

PDE12

PDK2

PDK3

PDP2

PDPN

PDSS2

PDZD8

PECR

PEMT

PERP

PET100

PEX5

PFDN2

PFDN4

PGR

PGRMC1

PHYKPL

PI4K2A

PI4KB

PID1

PIN1

PIN4

PINX1

PKM

PLA2G2A

PLA2G4A

PLA2G4B

PLA2G4C

PLA2G4F

PLA2G6

PLAUR

PLEKHN1

PLIN5

PLN

PMAIP1

PNKD

PNKP

PNPLA4

POLR1G

POU5F1

POU5F1B

PPA2

PPARGC1A

PPARGC1B

PPIF

PPM1B

PPM1E

PPP1CC

PPP1R13B

PPP1R15A

PPP2CA

PPP2CB

PPP2R1A

PPP2R2B

PPP3CA

PPP3CC

PPP3R1

PPP6C

PPRC1

PPTC7

PRDX5

PRDX6

PRELID1

PRELID2

PRELID3A

PRELID3B

PRIMPOL

PRKAA1

PRKACA

PRKCA

PRKCD

PRKCE

PRMT6

PRORP

PSEN1

PSEN2

PSMB4

PSMD10

PTCD1

PTCD2

PTEN

PTGES2

PTPMT1

PTPN1

PTPN11

PTS

PUS10

PYCARD

PYCR2

PYROXD2

QARS1

QRSL1

QTRT2

RAB11FIP3

RAB11FIP5

RAB29

RAB32

RAB38

RAB3A

RAB40AL

RAC2

RACK1

RAD51

RAD51C

RAF1

RANBP2

RAP1GDS1

RARS2

RB1CC1

REEP1

RFK

RGS2

RHBDD1

RHOT1

RHOT2

RHOU

RIDA

RIPK1

RMDN2

RMDN3

RMRP

RNASEL

RNASET2

RNF144B

RNF185

RNF186

RNF31

RNF41

RNF5

RPS27A

RPS3

RPS6KA6

RPS6KB1

RPUSD3

RPUSD4

RRM2B

RSAD1

RSAD2

RTL10

RTN4IP1

RXRA

S1PR4

SACS

SAE1

SARM1

SARS2

SCCPDH

SCP2

SDHA

SDHAF2

SDHAF3

SDHAF4

SDHB

SDHD

SDS

SECISBP2

SELENOO

SEPTIN4

SERAC1

SESN2

SFN

SFXN1

SFXN2

SFXN3

SFXN4

SFXN5

SGK1

SH3BP5

SH3GLB1

SHARPIN

SHC1

SHMT2

SIAH3

SIRT1

SIRT2

SIRT3

SIRT4

SIRT5

SIRT7

SLC11A2

SLC19A3

SLC22A4

SLC22A5

SLC25A1

SLC25A10

SLC25A13

SLC25A14

SLC25A15

SLC25A16

SLC25A19

SLC25A2

SLC25A20

SLC25A21

SLC25A22

SLC25A23

SLC25A24

SLC25A25

SLC25A27

SLC25A29

SLC25A3

SLC25A30

SLC25A31

SLC25A32

SLC25A33

SLC25A34

SLC25A35

SLC25A36

SLC25A39

SLC25A4

SLC25A43

SLC25A44

SLC25A45

SLC25A46

SLC25A47

SLC25A48

SLC25A5

SLC25A52

SLC25A6

SLC27A1

SLC27A3

SLC34A1

SLC35F6

SLC39A8

SLC3A1

SLC44A1

SLC44A2

SLC4A5

SLC8A1

SLC8A3

SLC8B1

SLC9A1

SLC9B2

SLIRP

SLIT3

SMCP

SMDT1

SMURF1

SNAP23

SNCA

SNN

SNPH

SOD1

SOD2

SORD

SOX10

SOX4

SP140

SPART

SPAST

SPATA18

SPATA19

SPATA5

SPATA7

SPHKAP

SPIRE1

SPNS1

SQSTM1

SRC

SREBF1

SREBF2

SRI

STAP1

STARD13

STARD7

STAT2

STING1

STK11

STMP1

STOM

STOML2

STOX1

STPG1

STX17

STXBP1

STYXL1

SUCLG2

SUGCT

SUOX

SUPV3L1

SURF1

SYBU

SYNE2

TACO1

TARDBP

TAT

TAZ

TBK1

TBRG4

TCAIM

TCHP

TDH

TDRD7

TERT

TEX10

TFAP2C

TFAP4

TFDP1

TFDP2

TFRC

TGM2

TH

THOP1

TICAM1

TIGAR

TIMM21

TIMM22

TIMM23B

TIMM29

TIMM44

TIMMDC1

TLE6

TMBIM6

TMEM102

TMEM135

TMEM14A

TMEM14B

TMEM14DP

TMEM14EP

TMEM186

TMEM71

TMEM8B

TMLHE

TMX2

TNFRSF1A

TNFSF10

TNRC18

TOMM20

TOMM34

TOMM7

TOMM70

TOP1MT

TOP3A

TP53

TP53AIP1

TP53BP2

TP63

TP73

TPPP

TRAF3

TRAF3IP3

TRAK1

TRAK2

TRAP1

TREM2

TRIAP1

TRIM14

TRIM31

TRIM39

TRMT10A

TRMT10B

TRMT5

TRMT61B

TRMU

TRNT1

TRUB1

TRUB2

TSC2

TSFM

TSPOAP1

TTC19

TTN

TUFM

TUSC2

TUSC3

TWNK

TXNIP

TXNRD3

TYMP

TYMS

UBA1

UBA52

UBB

UBC

UBE2D3

UBE2J2

UBE2L3

UBIAD1

UBL4B

UBL5

UCP1

UCP2

ULK1

UQCC1

UQCC3

UQCR10

UQCR11

UQCRB

UQCRC2

UQCRFS1

UQCRFS1P1

UQCRH

UQCRHL

UQCRQ

URI1

UROS

USP15

USP36

USP48

UXT

VAMP1

VASN

VAT1

VCP

VDAC1

VDAC3

VHL

VPS11

VPS13A

VPS13C

VPS13D

VPS35

VRK2

VWA8

WASF1

WDR26

WDR35

WDR45

WDR45B

WDR81

WDR93

WIPI1

WIPI2

WWOX

XAF1

XPC

XRCC3

YJEFN3

YKT6

YME1L1

YWHAB

YWHAE

YWHAG

YWHAH

YWHAQ

YWHAZ

ZBED3

ZBTB6

ZDHHC6

ZDHHC8

ZFYVE1

ZMIZ2

ZNF205

ZNF217

AADAT

AARS2

AASS

ABAT

ABCA9

ABCB10

ABCB6

ABCB7

ABCB8

ABCD1

ABCD2

ABCD3

ABHD10

ABHD11

ACAA1

ACACA

ACACB

ACAD10

ACAD11

ACAD8

ACAD9

ACADL

ACADM

ACADS

ACADSB

ACADVL

ACAT1

ACCS

ACLY

ACOT2

ACOT7

ACOT9

ACP6

ACSF2

ACSF3

ACSL1

ACSM3

ACSS3

ADCK1

ADCK2

ADCK5

ADCY10

ADHFE1

AFG1L

AGK

AGMAT

AGPAT4

AGPAT5

AGXT

AHCYL1

AIFM1

AIFM2

AK3

AKAP1

AKAP10

AKR7A2

ALAS1

ALDH1L1

ALDH1L2

ALDH2

ALDH3A2

ALDH4A1

ALDH7A1

ALDH9A1

ALKBH1

ALKBH7

AMT

ANGEL2

APOO

APOOL

ARF5

ARG2

ARL2

ARMCX1

ARMCX2

ARMCX3

ARMCX6

ATAD1

ATP23

ATP5F1A

ATP5F1D

ATP5IF1

ATP5MC1

ATP5MC2

ATP5MD

ATP5ME

ATP5MF

ATP5MF-PTCD1

ATP5MG

ATP5MPL

ATP5PB

ATP5PD

ATP5PF

ATPAF2

ATPSCKMT

AUH

BAD

BAK1

BAX

BCAT2

BCKDHA

BCKDHB

BCL2L10

BCL2L11

BCL2L13

BCL2L2

BCO2

BCS1L

BDH1

BID

BIK

BLOC1S1

BNIP3

BNIP3L

BOK

BOLA1

BOLA3

BPHL

C12orf65

C15orf48

C15orf61

C16orf91

C1QBP

C2orf69

C3orf33

C5orf63

C6orf136

C8orf82

CA5A

CA5B

CASP3

CASP8

CASP9

CBR3

CBR4

CCDC127

CCDC90B

CHCHD2

CHCHD3

CHCHD4

CHCHD5

CHDH

CHPT1

CISD1

CISD3

CKMT1A

CKMT2

CLPB

CLPP

CLYBL

CMC2

CMC4

CMPK2

COA1

COA6

COA7

COA8

COASY

COMT

COMTD1

COQ2

COQ6

COQ7

COQ8A

COX10

COX14

COX15

COX16

COX5A

COX5B

COX6A1

COX6A2

COX6B1

COX6B2

COX6C

COX7A1

COX7A2

COX7B

COX7B2

COX8A

COX8C

CPOX

CPT1A

CPT2

CRAT

CRLS1

CROT

CRY1

CRYZ

CS

CSKMT

CYB5B

CYB5R3

CYC1

CYCS

CYP11A1

CYP11B1

CYP11B2

CYP24A1

CYP27B1

D2HGDH

DAP3

DARS2

DBI

DCAKD

DCXR

DDX28

DELE1

DGLUCY

DGUOK

DHODH

DHRS1

DHRS2

DHRS4

DHRS7B

DHTKD1

DHX30

DIABLO

DLAT

DLD

DLST

DMAC1

DMAC2

DMAC2L

DMGDH

DMPK

DNA2

DNAJC28

DNAJC30

DNAJC4

DNLZ

DNM1L

DTYMK

DUS2

DUT

ECH1

ECHDC1

ECHDC3

ECI1

ECI2

ECSIT

ELAC2

ENDOG

EPHX2

ETFA

ETFRF1

ETHE1

EXOG

FABP1

FAHD1

FAHD2A

FAM136A

FAM185A

FAM210A

FAM210B

FARS2

FASN

FASTK

FASTKD1

FASTKD2

FDPS

FDX1

FDXR

FIS1

FKBP10

FKBP8

FLAD1

FMC1

FOXRED1

FTH1

GATC

GATD3A

GCDH

GFER

GFM2

GLDC

GLOD4

GLS

GLS2

GLUD1

GLYCTK

GPAM

GPAT2

GPD2

GPT2

GPX1

GPX4

GRHPR

GRPEL2

GSR

GSTK1

GTPBP6

GUK1

HADH

HADHB

HAO2

HARS2

HCCS

HDHD3

HDHD5

HEBP1

HIBADH

HIBCH

HINT1

HINT2

HINT3

HMGCL

HMGCS2

HOGA1

HPDL

HSCB

HSD17B10

HSD17B4

HSD17B8

HSDL1

HSDL2

HSPD1

HSPE1

HTATIP2

HTRA2

IARS2

IDH2

IDH3A

IDH3B

IDI1

ISCA1

ISCA2

ISOC2

IVD

KARS1

L2HGDH

LAP3

LDHB

LETM1

LETM2

LETMD1

LIAS

LIG3

LIPT2

LONP1

LYPLA1

LYPLAL1

LYRM1

LYRM2

LYRM4

LYRM7

LYRM9

MACROD1

MAIP1

MAOA

MAOB

MARCHF5

MARS2

MCAT

MCCC2

MCRIP2

MCU

MCUR1

MDH2

ME3

MECR

METAP1D

METTL15

METTL17

METTL4

METTL5

METTL8

MFF

MFN1

MFN2

MGME1

MGST1

MGST3

MICU1

MICU2

MIEF1

MIEF2

MIGA2

MIPEP

MLYCD

MMADHC

MMUT

MOCS1

MPST

MPV17

MPV17L

MPV17L2

MRM1

MRM2

MRPL1

MRPL11

MRPL12

MRPL13

MRPL14

MRPL15

MRPL16

MRPL17

MRPL2

MRPL22

MRPL23

MRPL27

MRPL28

MRPL3

MRPL30

MRPL33

MRPL34

MRPL35

MRPL36

MRPL37

MRPL38

MRPL39

MRPL4

MRPL40

MRPL41

MRPL42

MRPL43

MRPL44

MRPL45

MRPL46

MRPL47

MRPL48

MRPL49

MRPL50

MRPL53

MRPL54

MRPL55

MRPL57

MRPL9

MRPS10

MRPS11

MRPS12

MRPS14

MRPS15

MRPS16

MRPS18A

MRPS18C

MRPS2

MRPS21

MRPS22

MRPS23

MRPS24

MRPS25

MRPS26

MRPS27

MRPS31

MRPS33

MRPS5

MRPS6

MRPS7

MRPS9

MRRF

MRS2

MSRA

MSRB3

MTARC1

MTARC2

MT-ATP6

MT-ATP8

MTCH1

MT-CO3

MT-CYB

MTERF1

MTFMT

MTFP1

MTFR1

MTFR1L

MTFR2

MTG1

MTHFD2

MTHFD2L

MTHFS

MTIF2

MTIF3

MT-ND1

MT-ND2

MT-ND3

MT-ND4

MT-ND4L

MT-ND6

MTPAP

MTRES1

MTRF1

MTX1

MTX2

MUL1

MUTYH

MYG1

NADK2

NAGS

NARS2

NAT8L

NBR1

NDUFA1

NDUFA10

NDUFA11

NDUFA12

NDUFA13

NDUFA2

NDUFA4

NDUFA5

NDUFA6

NDUFA7

NDUFA8

NDUFA9

NDUFAB1

NDUFAF1

NDUFAF4

NDUFAF6

NDUFAF7

NDUFAF8

NDUFB2

NDUFB3

NDUFB6

NDUFB7

NDUFB8

NDUFB9

NDUFC1

NDUFC2

NDUFS1

NDUFS2

NDUFS3

NDUFS4

NDUFS7

NDUFV1

NDUFV2

NDUFV3

NEU4

NFU1

NGRN

NIF3L1

NIPSNAP3B

NIT1

NIT2

NLN

NLRX1

NME3

NME4

NME6

NMNAT3

NNT

NOA1

NOCT

NRDC

NSUN2

NSUN3

NSUN4

NT5DC2

NT5DC3

NUBPL

NUDT19

NUDT2

NUDT5

NUDT6

NUDT8

NUDT9

OAT

OCIAD1

OCIAD2

OGDH

OGDHL

OGG1

OMA1

OPA1

OPA3

OSBPL1A

OSGEPL1

OTC

OXA1L

OXCT2

OXLD1

OXNAD1

OXR1

OXSM

PABPC5

PAICS

PARK7

PARS2

PCBD2

PCCA

PCK2

PDE2A

PDF

PDHA1

PDHA2

PDHB

PDHX

PDK1

PDK4

PDP1

PDPR

PDSS1

PET117

PEX11B

PGAM5

PGS1

PHB

PHB2

PHYH

PICK1

PIF1

PIGBOS1

PINK1

PISD

PITRM1

PLD6

PLGRKT

PLPBP

PLSCR3

PMPCA

PMPCB

PNPLA8

PNPO

PNPT1

POLB

POLDIP2

POLG

POLG2

POLQ

POLRMT

PPM1K

PPOX

PRDX2

PRDX3

PRDX4

PREPL

PRKN

PRODH

PRODH2

PRSS35

PRXL2A

PTCD3

PTRH1

PTRH2

PUS1

PUSL1

PXMP2

PXMP4

PYCR1

PYURF

QDPR

QTRT1

RAB24

RAB5IF

RBFA

RCC1L

RDH13

RDH14

RECQL4

REXO2

RMDN1

RMND1

RNASEH1

ROMO1

RP11_469A15.2

RPIA

SAMM50

SARDH

SCO1

SCO2

SDHAF1

SDHC

SDR39U1

SDSL

SERHL2

SETD9

SLC25A11

SLC25A12

SLC25A18

SLC25A26

SLC25A28

SLC25A37

SLC25A38

SLC25A40

SLC25A41

SLC25A42

SLC25A51

SLC25A53

SLC30A9

SMIM20

SMIM8

SNAP29

SND1

SPATA20

SPG7

SPHK2

SPR

SPRYD4

SPTLC2

SQOR

SSBP1

STAR

SUCLA2

SUCLG1

SYNJ2BP

TAMM41

TARS2

TDRKH

TEFM

TFAM

TFB1M

TFB2M

THEM4

THEM5

THG1L

THNSL1

TIMM10

TIMM10B

TIMM13

TIMM17A

TIMM17B

TIMM23

TIMM50

TIMM8A

TIMM8B

TIMM9

TK2

TMEM11

TMEM126A

TMEM126B

TMEM143

TMEM14C

TMEM177

TMEM205

TMEM65

TMEM70

TOMM20L

TOMM22

TOMM40

TOMM40L

TOMM5

TOMM6

TRIT1

TRMT1

TRMT10C

TRMT2B

TSPO

TST

TSTD1

TSTD3

TXN2

TXNRD1

TXNRD2

UCP3

UNG

UQCC2

UQCRC1

USP30

VARS2

VDAC2

WARS2

XPNPEP3

YARS2

YBEY

YRDC

ZADH2

**Table S2 Mitochondrial gene set**

**
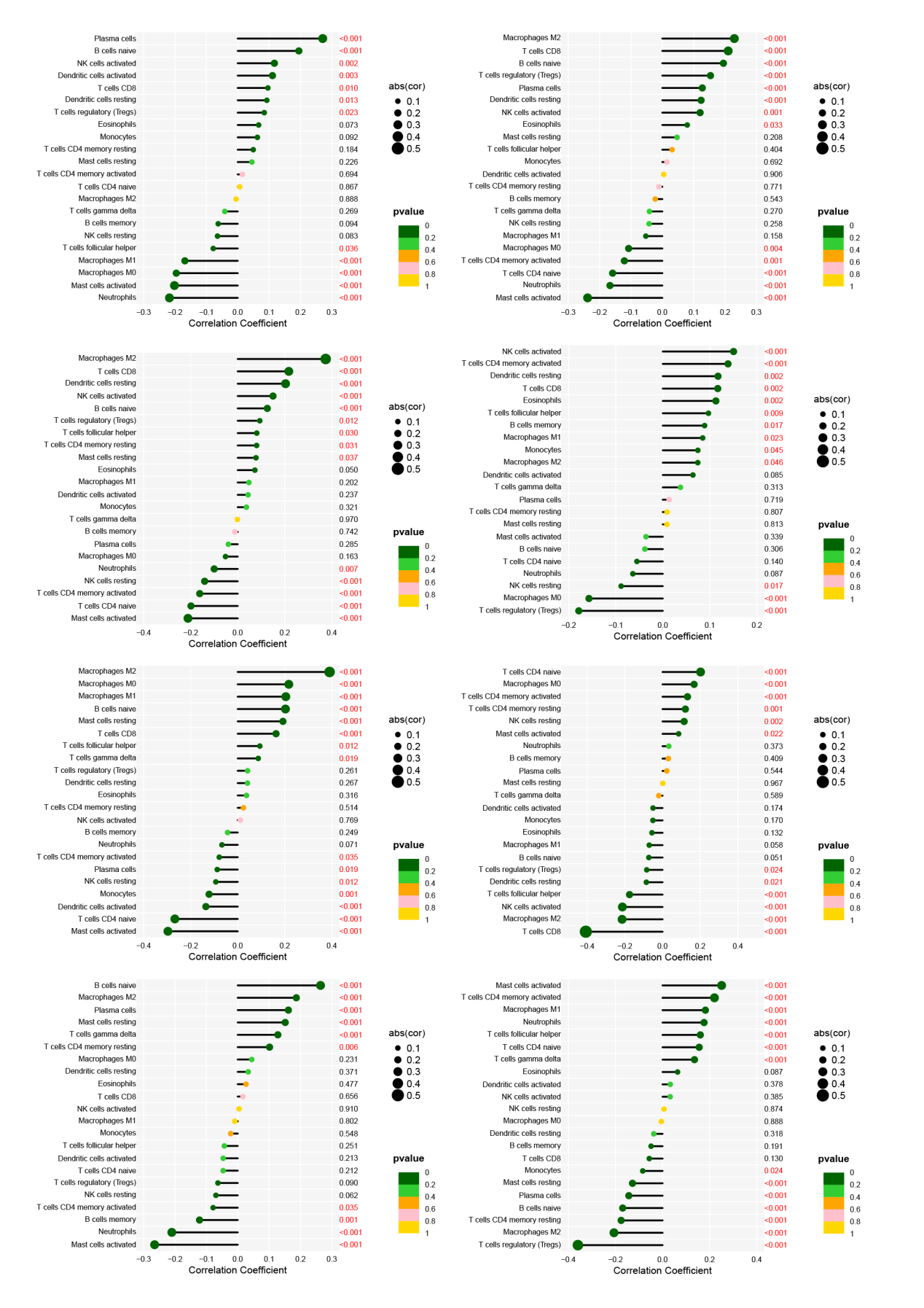
**

**Figure S3 Correlation analysis between immune infiltration results and eight core genes**

Gene Type

CCL3 Chemokine

CCL4 Chemokine

CCL8 Chemokine

CXCL10 Chemokine

CCL26 Chemokine

CCL7 Chemokine

CCL13 Chemokine

CCR5 Receptor

CXCL11 Chemokine

CCL18 Chemokine

CCL5 Chemokine

CCL15 Chemokine

CCR1 Receptor

CXCL9 Chemokine

CXCL16 Chemokine

CXCL5 Chemokine

CXCL1 Chemokine

CCL2 Chemokine

CXCL2 Chemokine

CXCL3 Chemokine

CCL24 Chemokine

CCL23 Chemokine

CXCL13 Chemokine

CXCR6 Receptor

CCR7 Receptor

CXCL8 Chemokine

CCL20 Chemokine

CXCL12 Chemokine

CCL11 Chemokine

CXCR3 Receptor

CCR2 Receptor

XCL2 Chemokine

CXCL17 Chemokine

CXCR4 Receptor

CCL17 Chemokine

CXCR2 Receptor

CCL21 Chemokine

CXCR1 Receptor

CCL28 Chemokine

CCR8 Receptor

CX3CL1 Chemokine

CXCL6 Chemokine

CXCL14 Chemokine

CCL25 Chemokine

CCR3 Receptor

XCR1 Receptor

CCL1 Chemokine

CCL19 Chemokine

CCL22 Chemokine

CCR4 Receptor

CCR10 Receptor

CCR6 Receptor

CX3CR1 Receptor

CCL14 Chemokine

XCL1 Chemokine

CCR9 Receptor

CXCR5 Receptor

CCL16 Chemokine

CCL27 Chemokine

TAP2 MHC

TAP1 MHC

HLA-DRB1 MHC

HLA-DQB1 MHC

HLA-DRA MHC

HLA-DPA1 MHC

HLA-DPB1 MHC

HLA-DQA1 MHC

HLA-DMB MHC

HLA-F MHC

HLA-A MHC

HLA-B MHC

B2M MHC

HLA-DOB MHC

TAPBP MHC

HLA-E MHC

HLA-C MHC

HLA-DQA2 MHC

HLA-DOA MHC

HLA-G MHC

HLA-DMA MHC

TNFSF9 Immunostimulator

CD86 Immunostimulator

TNFRSF18 Immunostimulator

TNFSF13B Immunostimulator

PVR Immunostimulator

IL2RA Immunostimulator

CD80 Immunostimulator

CD70 Immunostimulator

NT5E Immunostimulator

IL6 Immunostimulator

TNFRSF9 Immunostimulator

TNFSF14 Immunostimulator

ICOS Immunostimulator

TNFSF15 Immunostimulator

TNFSF4 Immunostimulator

TNFRSF8 Immunostimulator

CD48 Immunostimulator

TNFRSF4 Immunostimulator

ENTPD1 Immunostimulator

IL6R Immunostimulator

TNFRSF14 Immunostimulator

CD276 Immunostimulator

MICB Immunostimulator

LTA Immunostimulator

CD28 Immunostimulator

CD27 Immunostimulator

KLRC1 Immunostimulator

ULBP1 Immunostimulator

TNFSF18 Immunostimulator

BTNL2 Immunostimulator

ICOSLG Immunostimulator

RAET1E Immunostimulator

HHLA2 Immunostimulator

TNFSF13 Immunostimulator

CD40 Immunostimulator

TNFRSF25 Immunostimulator

TNFRSF17 Immunostimulator

CD40LG Immunostimulator

KLRK1 Immunostimulator

TNFRSF13B Immunostimulator

TMIGD2 Immunostimulator

TNFRSF13C Immunostimulator

**Table S4 122 immune target gene sets**

Including Immunostimulators, Chemokines, Receptors, and MHC sites.

**
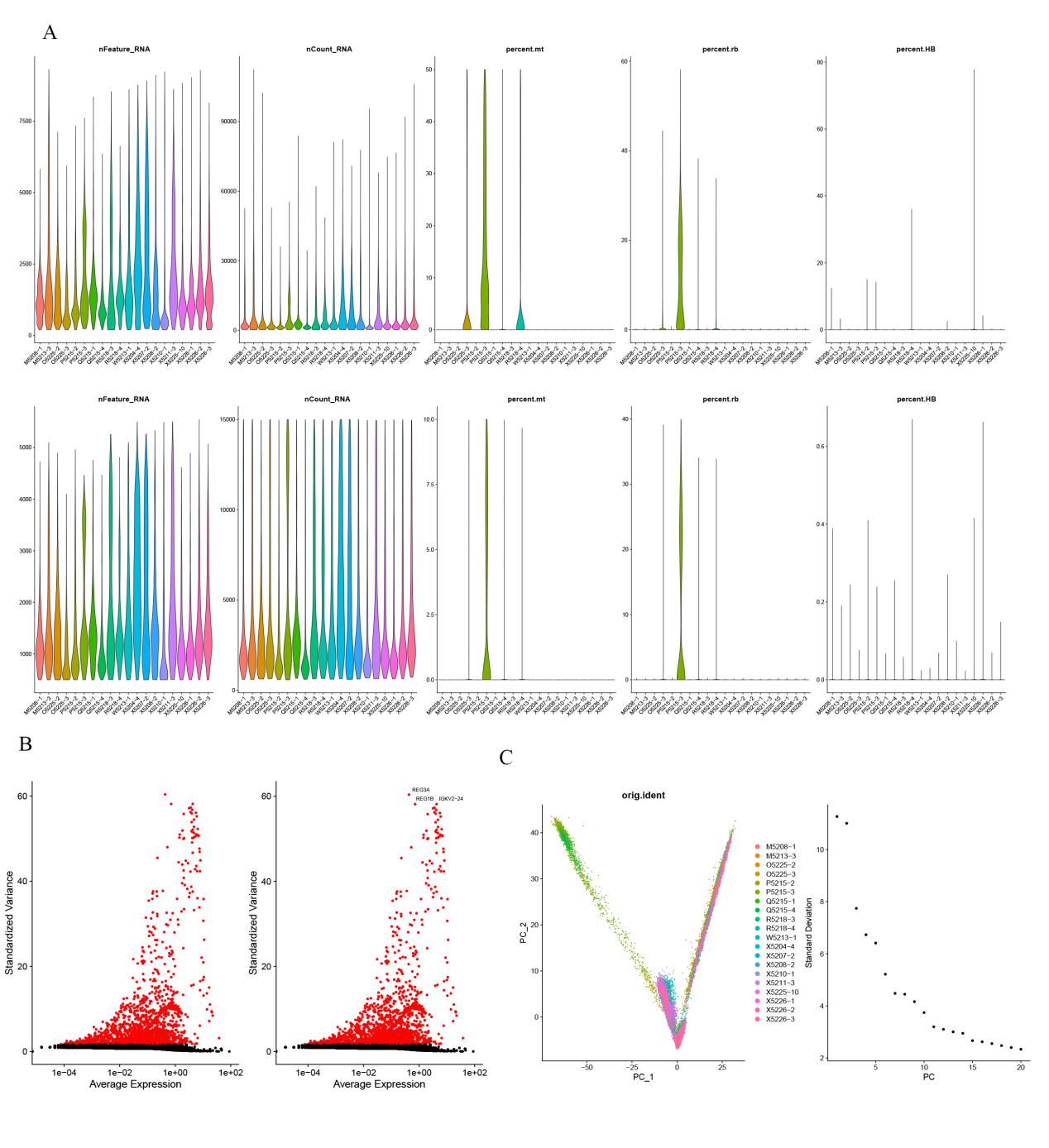
**

**Figure S5 Single-cell data quality control**

(A)Before and after quality control of single-cell datasets. (B)Single-cell data hypervariable genest. (C)PCA results of single-cell dataset.

Apoptosis

AATF

ABL1

ACAA2

ACKR3

ACVR1

ACVR1B

ADORA1

AEN

AGT

AGTR2

AIFM1

AKT1

ANXA6

APAF1

APPL1

AR

ARHGEF2

ARL6IP5

ARMC10

ARRB2

ASAH2

ATF3

ATF4

ATM

ATP2A1

ATP2A3

ATP5IF1

AVP

BAD

BAG3

BAG5

BAG6

BAK1

BAX

BBC3

BCAP31

BCL10

BCL2

BCL2A1

BCL2L1

BCL2L10

BCL2L11

BCL2L12

BCL2L14

BCL2L2

BCL3

BCLAF1

BDKRB2

BDNF

BECN1

BID

BIK

BIRC6

BLOC1S2

BMF

BMP4

BMP5

BMPR1B

BNIP3

BNIP3L

BOK

BRCA1

BRCA2

BRSK2

BTK

CAAP1

CASP1

CASP10

CASP12

CASP2

CASP3

CASP4

CASP5

CASP8

CASP8AP2

CASP9

CAV1

CCAR2

CCK

CD14

CD24

CD27

CD28

CD38

CD3E

CD44

CD5

CD70

CD74

CDIP1

CDKN1A

CDKN2D

CEBPB

CFLAR

CHAC1

CHCHD10

CHEK2

CIB1

CIDEB

CLU

COA8

COL2A1

CRADD

CREB3

CREB3L1

CRH

CRIP1

CSF2

CSNK2A1

CSNK2A2

CTH

CTNNA1

CTSC

CTTN

CUL1

CUL2

CUL3

CUL4A

CUL5

CX3CL1

CX3CR1

CXCL12

CYLD

CYP1B1

DAB2IP

DAP

DAP3

DAPK1

DAPK2

DAPK3

DAPL1

DAXX

DBH

DCC

DDIAS

DDIT3

DDIT4

DDX3X

DDX47

DDX5

DEDD

DEDD2

DELE1

DEPTOR

DIABLO

DIDO1

DNAJA1

DNAJC10

DNM1L

DPF2

DYRK2

E2F1

E2F2

EDA2R

EIF2AK3

ELL3

ENO1

EP300

EPHA2

EPO

ERBB3

ERCC6

ERN1

ERN2

ERO1A

ERP29

EYA1

EYA2

EYA3

EYA4

FADD

FAF1

FAIM

FAIM2

FAM162A

FAS

FASLG

FASTK

FBH1

FBXW7

FEM1B

FGA

FGB

FGF10

FGFR1

FGFR3

FGG

FHIT

FIGNL1

FIS1

FNIP2

FXN

FYN

FZD9

G0S2

GABARAP

GATA1

GATA4

GCLM

GDNF

GFRAL

GGCT

GHITM

GNAI2

GNAI3

GPER1

GPX1

GRINA

GSDME

GSK3A

GSK3B

GSKIP

GSTP1

GZMB

HDAC1

HERPUD1

HGF

HIC1

HIF1A

HINT1

HIP1

HIP1R

HIPK1

HIPK2

HMGB2

HMOX1

HNRNPK

HRAS

HRK

HSPA1A

HSPA1B

HSPB1

HTRA2

HTT

HYAL2

HYOU1

ICAM1

IFI16

IFI27

IFI27L1

IFI27L2

IFI6

IFNB1

IFNG

IGF1

IKBKE

IL12A

IL19

IL1A

IL1B

IL2

IL20RA

IL33

IL4

IL6R

IL7

INCA1

ING2

ING5

INHBA

INHBB

INS

ITGA6

ITGAM

ITGAV

ITM2C

ITPR1

ITPRIP

IVNS1ABP

JAK2

JMY

JUN

KDM1A

KITLG

KRT18

KRT8

LCK

LGALS12

LGALS3

LRRK2

LTBR

LY96

MADD

MAEL

MAGEA3

MAP2K5

MAP3K5

MAPK7

MAPK8

MAPK8IP1

MAPK8IP2

MAPK9

MARCHF7

MAZ

MCL1

MDM2

MELK

MFF

MIF

MIR132

MIR15A

MIR16-1

MIR17

MIR198

MIR21

MIR210

MIR221

MIR222

MIR26B

MIR27B

MIR449A

MKNK2

MLH1

MLLT11

MMP9

MNT

MOAP1

MPV17L

MSH2

MSH6

MSX1

MUC1

MUL1

MYBBP1A

NACC2

NANOS3

NBN

NCK1

NCK2

NDUFA13

NDUFS3

NFATC4

NFE2L2

NGF

NGFR

NKX3-1

NLE1

NME5

NMT1

NOC2L

NOG

NOL3

NONO

NOS3

NOX1

NR4A2

NUPR1

OPA1

P2RX4

P2RX7

P4HB

PAK2

PAK5

PARK7

PARP1

PARP2

PAWR

PCGF2

PDCD10

PDCD5

PDCD6

PDIA3

PDK1

PDK2

PDPK1

PDX1

PEA15

PELI3

PERP

PF4

PHIP

PHLDA3

PIAS4

PIDD1

PIH1D1

PIK3R1

PINK1

PLAGL2

PLAUR

PLEKHF1

PLSCR3

PMAIP1

PML

POLB

POU4F1

POU4F2

PPARD

PPIA

PPIF

PPM1F

PPP1CA

PPP1R13B

PPP1R15A

PPP2R1B

PPP3CC

PPP3R1

PRDX2

PRELID1

PRKCA

PRKCD

PRKDC

PRKN

PRKRA

PRODH

PSEN1

PSMD10

PSME3

PTEN

PTGIS

PTH

PTPMT1

PTPN1

PTPN2

PTPRC

PTTG1IP

PYCARD

QARS1

RACK1

RAF1

RB1

RB1CC1

RBCK1

RELA

RET

RFFL

RHOT1

RHOT2

RIPK1

RIPK3

RNF183

RNF186

RNF34

RNF41

RPL11

RPL26

RPS27L

RPS3

RPS6KB1

RPS7

RRP8

RTKN2

RTL10

S100A8

S100A9

SCG2

SCN2A

SCRT2

SELENOK

SELENOS

SENP1

SEPTIN4

SERINC3

SERPINE1

SFN

SFPQ

SFRP1

SFRP2

SGMS1

SGPL1

SGPP1

SH3RF1

SHH

SHISA5

SIAH1

SIAH2

SIRT1

SIVA1

SKIL

SLC25A5

SLC35F6

SLC9A3R1

SMAD3

SNAI1

SNAI2

SNW1

SOD1

SOD2

SORT1

SP100

SRC

SRPX

SST

SSTR3

ST20

STK11

STK24

STK25

STK3

STK4

STRADB

STX4

STYXL1

SYVN1

TAF9

TAF9B

TCF7L2

TERT

TFDP1

TFDP2

TFPT

TGFB1

TGFB2

TGFBR1

THBS1

TICAM1

TICAM2

TIMM50

TIMP3

TLR3

TLR4

TM2D1

TMBIM1

TMBIM6

TMC8

TMEM102

TMEM109

TMEM117

TMEM14A

TMEM161A

TNF

TNFAIP3

TNFRSF10A

TNFRSF10B

TNFRSF10C

TNFRSF12A

TNFRSF1A

TNFRSF1B

TNFRSF25

TNFSF10

TNFSF12

TOPORS

TP53

TP53BP2

TP63

TP73

TPD52L1

TPT1

TRADD

TRAF1

TRAF2

TRAF7

TRAP1

TRIAP1

TRIB3

TRIM32

TRIM39

TXNDC12

TYROBP

UACA

UBB

UBE2K

UBE4B

UBQLN1

UMOD

UNC5B

URI1

USP28

USP47

VDAC2

VNN1

WDR35

WNT4

WWOX

XBP1

YAP1

YBX3

YWHAB

YWHAE

YWHAG

YWHAH

YWHAQ

YWHAZ

ZC3HC1

ZDHHC3

ZMYND11

ZNF205

ZNF385A

ZNF385B

ZNF622

ZSWIM2

**Table S6 Apoptosis gene set**

**
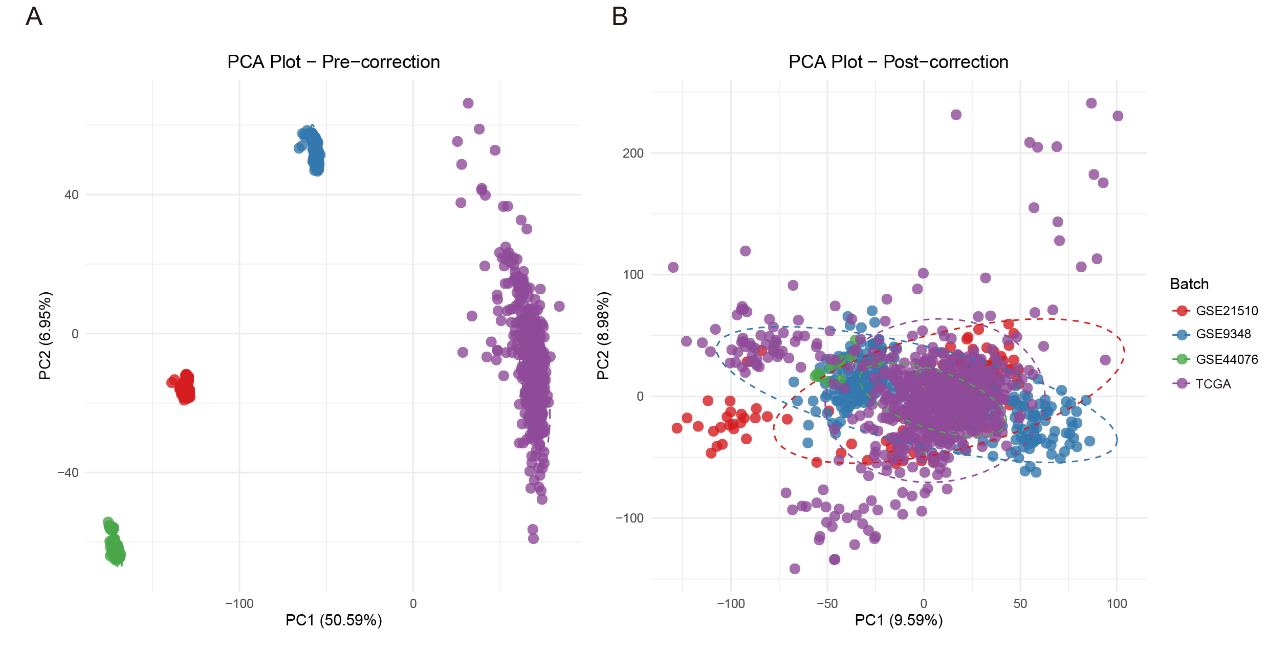
**

**Figure S7 PCA plot**

PCA diagrams before and after batch correction to observe the effect of batch removal.

**
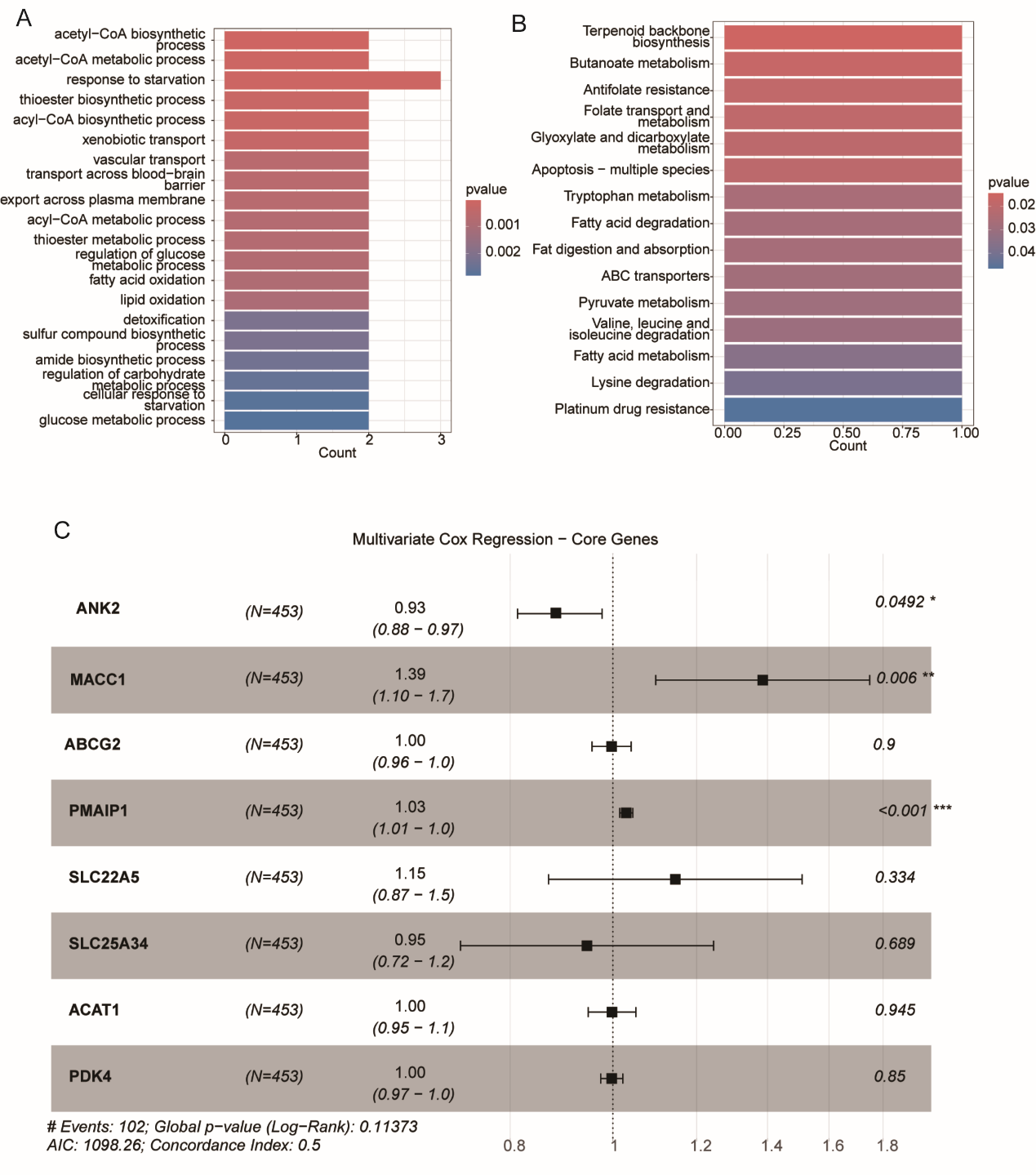
**

**Figure S8**

(A)Go analysis. (B) KEGG analysis. (C)Multivariate Cox Regression. (*P<0.05,**P<0.01,***P<0.001)

**
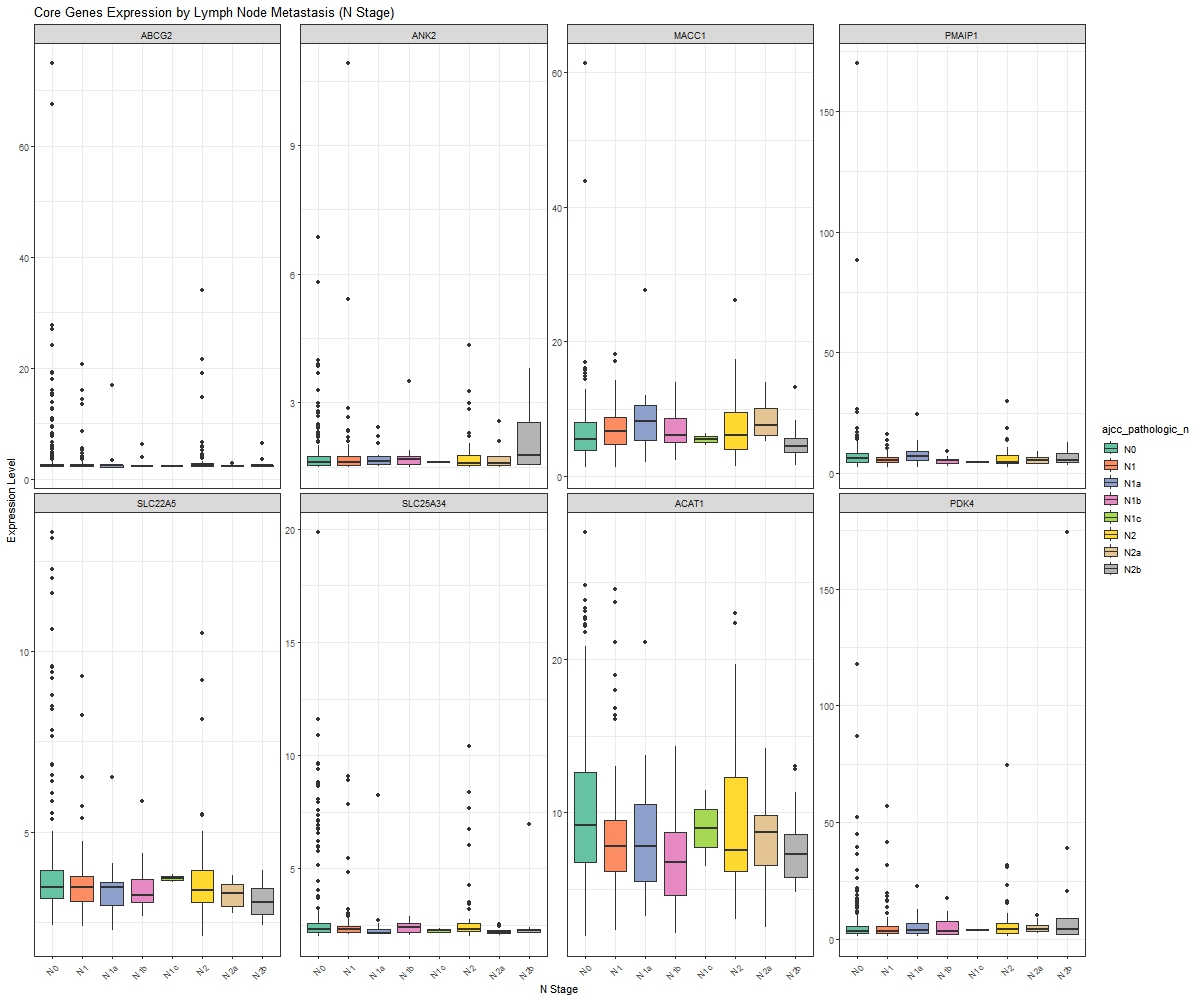
**

**Figure S9 N stage correlation analysis**

**
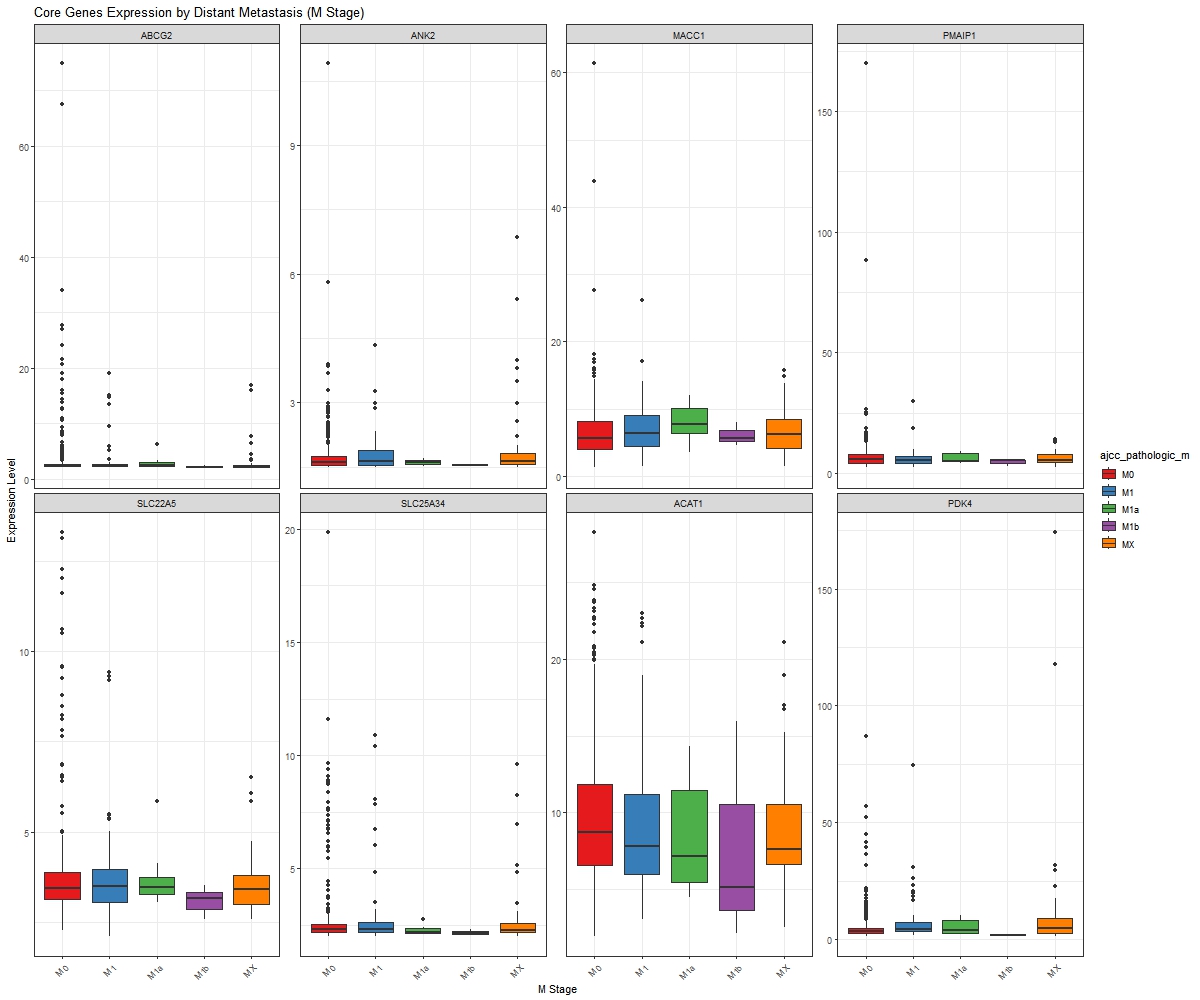
**

**Figure S10 M stage correlation analysis**
